# Polymeric carbohydrates utilization separates microbiomes into niches: insights into the diversity of microbial carbohydrate-active enzymes in the inner shelf of the Pearl River Estuary, China

**DOI:** 10.3389/fmicb.2023.1180321

**Published:** 2023-06-21

**Authors:** Cui-Ci Sun, Wen-Jie Zhao, Wei-Zhong Yue, Hao Cheng, Fu-Lin Sun, Yu-Tu Wang, Mei-Lin Wu, Anja Engel, You-Shao Wang

**Affiliations:** ^1^State Key Laboratory of Tropical Oceanography, South China Sea Institute of Oceanology, Chinese Academy of Sciences, Guangzhou, China; ^2^Daya Bay Marine Biology Research Station, Chinese Academy of Sciences, Shenzhen, China; ^3^University of Chinese Academy of Sciences, Beijing, China; ^4^GEOMAR Helmholtz Centre for Ocean Research Kiel, Kiel, Germany

**Keywords:** microbial CAZyme diversity, metagenomics analysis, glycan niche, the Pearl River Estuary, microbiome

## Abstract

Polymeric carbohydrates are abundant and their recycling by microbes is a key process of the ocean carbon cycle. A deeper analysis of carbohydrate-active enzymes (CAZymes) can offer a window into the mechanisms of microbial communities to degrade carbohydrates in the ocean. In this study, metagenomic genes encoding microbial CAZymes and sugar transporter systems were predicted to assess the microbial glycan niches and functional potentials of glycan utilization in the inner shelf of the Pearl River Estuary (PRE). The CAZymes gene compositions were significantly different between in free-living (0.2–3 μm, FL) and particle-associated (>3 μm, PA) bacteria of the water column and between water and surface sediments, reflecting glycan niche separation on size fraction and selective degradation in depth. Proteobacteria and Bacteroidota had the highest abundance and glycan niche width of CAZymes genes, respectively. At the genus level, *Alteromonas* (Gammaproteobacteria) exhibited the greatest abundance and glycan niche width of CAZymes genes and were marked by a high abundance of periplasmic transporter protein TonB and members of the major facilitator superfamily (MFS). The increasing contribution of genes encoding CAZymes and transporters for *Alteromonas* in bottom water contrasted to surface water and their metabolism are tightly related with particulate carbohydrates (pectin, alginate, starch, lignin-cellulose, chitin, and peptidoglycan) rather than on the utilization of ambient-water DOC. *Candidatus Pelagibacter* (Alphaproteobacteria) had a narrow glycan niche and was primarily preferred for nitrogen-containing carbohydrates, while their abundant sugar ABC (ATP binding cassette) transporter supported the scavenging mode for carbohydrate assimilation. Planctomycetota, Verrucomicrobiota, and Bacteroidota had similar potential glycan niches in the consumption of the main component of transparent exopolymer particles (sulfated fucose and rhamnose containing polysaccharide and sulfated-N-glycan), developing considerable niche overlap among these taxa. The most abundant CAZymes and transporter genes as well as the widest glycan niche in the abundant bacterial taxa implied their potential key roles on the organic carbon utilization, and the high degree of glycan niches separation and polysaccharide composition importantly influenced bacterial communities in the coastal waters of PRE. These findings expand the current understanding of the organic carbon biotransformation, underlying the size-fractionated glycan niche separation near the estuarine system.

## 1. Introduction

Carbohydrates constitute a large fraction of labile and semi-labile organic matter and mainly derive from phytoplankton in the ocean (Pakulski and Benner, 1994). Much of the total carbohydrate fraction is combined in oligo-and polysaccharides (Benner, 2002; Myklestad and Børsheim, 2007; Sperling et al., 2017), and polysaccharides (i.e., glycans) can contribute up to 50% to total phytoplankton biomass (Painter, 1983; Becker et al., 2020). Carbohydrate degradation in the ocean is mainly driven by prokaryotic organisms. Labile polysaccharides are rapidly degraded and respired. Polysaccharides in sinking particles are remineralized while sinking through the water column or in sediments where the more recalcitrant fraction may accumulate (Jiao et al., 2011; Arnosti et al., 2021). Thus, particulate polysaccharides can contribute substantially to carbon export from surface waters (Vidal-Melgosa et al., 2021).

Polysaccharides are chemically diverse and contain various structures and highly branched molecules. CAZymes are used by heterotrophic microbes to assemble, break down, and modify glycans and glycoconjugates. In general, the diversity of CAZymes can mirror the substrates that they utilize and the ecological and/or biogeochemical processes in the ocean (Teeling et al., 2012, 2016; Zhao et al., 2020; Baltar et al., 2021). In addition, long-term investigations carried out by Teeling et al. (2016) revealed that variations of CAZymes genes traits and the accompanying succession of bacterial compositions are largely governed by deterministic principles such as substrate-induced forcing. Furthermore, Vidal-Melgosa et al. (2021) found that the abundance of CAZymes in the North Sea of the Atlantic Ocean involved in the degradation of laminarin was much higher than fucoidan during the outbreak of diatom bloom, resulting in the carbon sequestration in the form of fucoidan (Vidal-Melgosa et al., 2021). For alginate and pectin, identified alginate-degrading enzymes and polysaccharide utilization loci (PULs) in metagenome-assembled genomes related to Alteromonadacea and Bacteroidetes in seawater indicated that these CAZyme gene pools are not phylogenetically widespread but niche specialized (Hehemann et al., 2017; Thomas et al., 2021; Wolter et al., 2021). Bacteroidota, Planctomycetota, and Verrucomicrobiota phylum are prominent for the degradation of a wide range of complex carbohydrates (i.e., plant cell wall, bacterial EPS, and sulfated glycan) due to their high diversity of CAZymes and sulfatases, which improve their adaptability in many diverse environments (Costa et al., 2020; Sichert et al., 2020; Luis et al., 2022).

Niche is a complex description of how a microbial species uses its environment. For a long time, less attention has been paid to the ecological niche concept of microbiome than to other plants and animals. Microbial niche breadth has been measured for specific aspects of the environment [e.g., temperature (Sauer et al., 2015), pH (Kuang et al., 2013)] and nutrient availability (Kits et al., 2017; Herold et al., 2020; Dal Bello et al., 2021). Recently, von Meijenfeldt et al. (2023) used social niche breadth score to reveal niche range strategies of generalists and specialists, based on the variability of the communities with which it associates. Since metabolic traits (such as the ability to metabolize certain substrates or synthesize molecules) are the most important factors affecting the niche of microbial cells (Garza et al., 2018; Fahimipour and Gross, 2020; Dal Bello et al., 2021), and the polymers of carbohydrates represent a large pool of organic matter in the ocean, CAZymes diversity, glycan niche width and the related measurement of glycan overlap based on the CAZymes genes distribution in the microbial community are presumably closely related to microbial utilization of the organic carbon matter in the environment and their niche specifications (Teeling et al., 2012, 2016; Smits et al., 2017; Avci et al., 2020; Dal Bello et al., 2021).

Besides CAZymes, outer membrane transporters specific for polysaccharide uptake, such as the SusC-like TonB-dependent transporter (TBDT) (starch utilization system), also can serve as an indicator for estimating bacterial polysaccharide utilization (Francis et al., 2021). Recently, according to a new model of the strategy of microbial processing polysaccharides degradation production, marine microbes were simplified into three types: sharer, scavenger, and selfish bacteria (Reintjes et al., 2019). Sharers are bacteria that secrete extracellular enzymes and hydrolyze substrates in the external environment; oppositely, scavengers cannot produce corresponding enzymes for high molecular weight polysaccharides and are considered to benefit from hydrolysis products (e.g., oligosaccharide) degraded by sharers. The selfish bacteria are defined as hydrolyzing substrates with little diffusive loss (Reintjes et al., 2019; Arnosti et al., 2021). In general, the selfish bacteria have abundant cell-associated enzymes and transporters in outer membranes (e.g., SusC), which enable the hydrolysates quickly come into the periplasm space instead of being utilized by other bacteria (Cuskin et al., 2015). The insights from bacterial CAZymes diversity contribute to the understanding of algae-bacteria interactions and the remineralization of polysaccharides in the light of large amounts of algae primary productivity on global scales. So far, polysaccharide-degrading capabilities of marine bacteria *in situ* driven by CAZymes are largely unknown (Lazar et al., 2016; Orsi et al., 2018).

Estuaries and adjacent waters with enhanced nutrient concentrations are typically highly productive and show elevated polysaccharide concentrations including algal storage polysaccharides (β-1,3-glucans such as laminarin; α-1,4-glucans such as starch and glycogen) (Alderkamp et al., 2007), and cell matrix and cell wall constituents of both algae and other organisms (cellulose or hemicellulose, xylose, lignin, peptidoglycan, pectin, and chitin) (Okuda, 2002; Tremblay and Benner, 2006). Moreover, transparent exopolymer particles (TEP) are ubiquitous in estuarine systems. These gel-like particles are rich in acidic polysaccharides, which include fucose- and rhamnose-rich sulfated heteropolysaccharides (sulfate half-ester) or uronic acid-containing polysaccharides with carboxyl groups, such as alginate and pectin (Hung et al., 2001; Passow, 2002; Sperling et al., 2017). In addition, terrestrial higher plants are rich in lignin and cellulose and are often exported to estuaries and adjacent coastal seas (Benner and Kaiser, 2011). In the transition zone from the estuary and shelf to the continent, carbohydrates undergo significant degradation and transformation processes (Benner, 2004). Accordingly, bacterial CAZymes perform complex functions and are responsible for much of the carbon turnover in the estuary, characterized by high loads of organic carbon. Yet, little is known about the diversity of microbial CAZymes and their role in polysaccharide decomposition in the estuarine system (Baker et al., 2015; Smith et al., 2019).

In this study, we sampled the inner shelf of the Pearl River Estuary, where the contribution of terrigenous organic carbon was on average 34 ± 4% while polysaccharides and proteinaceous matter were mainly derived from phyto- and bacterioplankton (He et al., 2010a,b; Zhang and Ran, 2014; Sun et al., 2022). In addition, TEP was an important particulate component of the total organic pool in the PRE (Sun et al., 2012). A recent study found that the particle-associated bacteria communities in the PRE differed from free-living bacteria compositions (Liu Y. Y. et al., 2020) and showed higher connectivity between bacterioplankton and archaea plankton communities than free-living bacteria (Wang et al., 2020).

To reveal the microbial consortia involved in the transformation of functionally different polysaccharides, genes of microbial CAZymes, sugar transporter, sulfatase, and their functional potentials of polysaccharide utilization were predicted for different size fractions, representing free-living bacteria (0.2–3 μm) and particle-associated bacteria (>3 μm) in water and surface sediments of the inner PRE based on metagenomic data. We assessed microbial CAZymes diversity, glycan niche width, glycan specificities, and metabolic strategies for implying organic carbon turnover in the inner shelf of the estuarine system.

## 2. Sampling and method

### 2.1. Sampling

**Figure 1:**
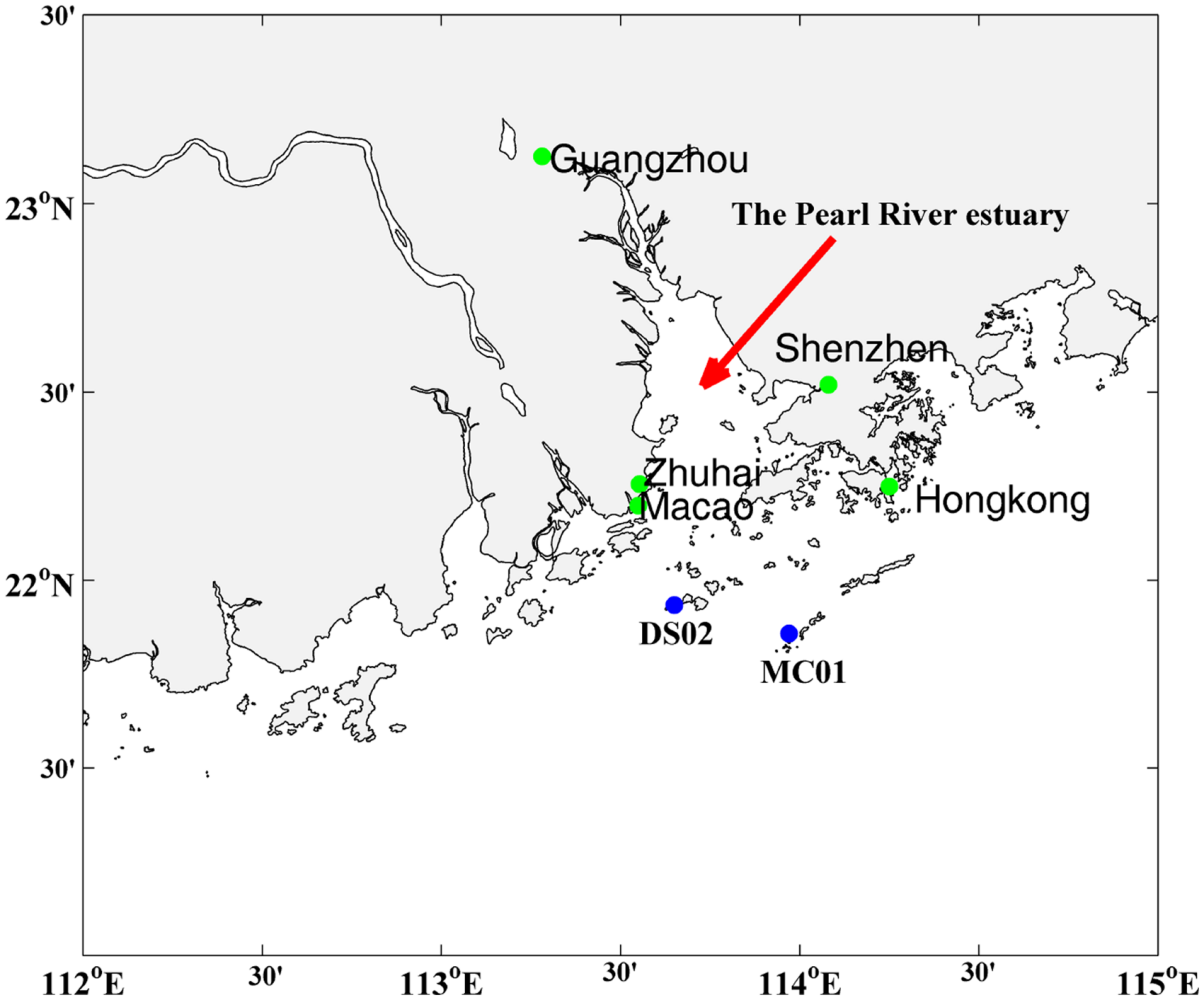
Sampling stations in the inner shelf of the Pearl River estuary.

The study was conducted on the inner shelf of the PRE in Nov 2020. The sampling sites are shown in Figure 1. Water samples were collected near the surface (0.5 m depth) and bottom layers, using 5.0-L Niskin bottles. 2 L of seawater was filtered first through 3-μm-pore-size and subsequently through 0.22-μm-pore-size filter membranes (Millipore, Bedford, United States) to collect particle-associated (>3 μm) and free-living bacteria (0.2–3 μm), respectively. The surface sediment (upper 1–2 cm) was collected using a grab sampler. The filter membranes and surface sediment (15 g) were frozen immediately in liquid nitrogen before being stored at −80°C until DNA extraction. Salinity, pH, turbidity, and temperature were measured *in situ* using a YSI 6600 probe (YSI Inc., United States).

### 2.2. Chemical analysis

For particulate organic carbon (POC) and Chl*a*, 1 L of seawater was filtered through pre-combusted (10 h, 450°C) glass fiber filters (0.7 μm pore size, Whatman, USA) under a gentle vacuum of <150 mm Hg. From each sample, 30 mL of filtrate was collected into 40 mL pre-combusted glass vials and immediately stored at-20°C for dissolved organic carbon (DOC) analysis. 200 mL of filtered seawater from each depth were stored at −20°C for nutrient analysis (nitrate, nitrite, ammonium salt, phosphate, and silicate). Chl*a* concentrations were determined after extraction in 90% acetone overnight (Parsons et al., 1984). The concentration of POC was determined with a PE2400 Series II CHNS/O analyzer (PerkinElmer, USA). DOC concentration was measured using a Shimadzu TOC-V analyzer (Shimadzu Inc., Japan) (Sun et al., 2012). Nutrients were determined using a four-channel continuous flow Technicon AA3 auto-analyzer (Bran+Luebbe GmbH, Germany), and the detection limits were about 0.04 μmol L^−1^ for inorganic nitrogen nutrients and 0.03 μmol L^−1^ for phosphate and silicate, respectively.

For TOC in sediment, samples were oven-dried at 50°C, ground, and homogenized with a pestle and mortar, then passed through a mesh sieve (250 μm in pore size). Sediment samples were soaked in 1 mol L^−1^ HCl at room temperature for 24 h (Prahl et al., 1994), rinsed with Milli-Q water to remove salts, and then oven-dried at 50°C. Then, TOC was detected using dry combustion with a Perkin-Elmer 2400 CHNS/O analyzer. The relative standard deviations (RSDs) of TOC determinations were < 5%. The content of Acid Volatile Sulfide (AVS) in sediments was measured with the methylene-blue-spectrophotometric method according to the standard method of the People’s Republic of China (HJ 833-2017). Then, 3.0 g of wet sample was put into the nitrogen-purging meter (Taipute Co. Ltd., LHW-6A) at first, and (1 + 2) HCl was dripped in. H_2_S produced in the nitrogen-purging meter was absorbed by the mixed solution of zinc acetate and sodium acetate. Finally, the content of AVS in the mixed solution was measured with the UV spectrophotometer (Shimadzu, UV-1700).

### 2.3. DNA preparation, sequencing, metagenome assembly, and analysis

Sample DNA was extracted in triplicate from 0.5 g of fresh surface sediment and filtered samples using the Fast DNA spin kit (MP Biomedicals, Cleveland, United States) following the manufacturer’s instructions. The quality and integrity of the DNA extracts were assessed using a NanoDrop 2000 spectrophotometer. The extracted microbial DNA was processed to construct metagenome shotgun sequencing libraries with insert sizes of 350 bp using the Illumina TruSeq Nano DNA LT Library Preparation Kit. Each library was sequenced by an Illumina HiSeq X-ten platform (2 × 150; Illumina, USA) at Nuohe Biotechnology Co., Ltd. (Tianjin, China). The sequences can be found on the National Center for Biotechnology Information (NCBI), with the accession number PRJNA859198.

Metagenomic raw reads were quality-checked with FASTQC v0.11.8 (Davis et al., 2013) and the evaluation of clean reads quality was trimmed with a sliding window approach using Trimmomatic (Bolger et al., 2014). Trimmed reads were processed and assembled using MEGAHIT with default settings (Bankevich et al., 2012); Open reading frames (ORFs) were identified by the Prodigal software (Hyatt et al., 2010) and further annotated for function and taxa identification (Buchfink et al., 2015). Filtered reads were mapped to the ORFs using Bowtie2 to calculate the abundance of each ORF (Langmead and Salzberg, 2012). Taxonomic classifications of the metagenomic sequencing reads from each sample were performed against the NR database (non-redundant proteins database, downloaded from ftp://ftp.ncbi.nlm.nih.gov/blast/db), which included proteins from archaea, bacteria, viruses, and fungi.

Annotation of CAZymes was done using dbCAN v10 against the CAZy database v09242021based on hmmer filter (Zhang et al., 2018) and additionally confirmed using DIAMOND BLAST (Buchfink et al., 2015), and CAZymes were only analyzed for contigs >500 bp. Sulfatases were predicted with Blast v2.10.1 against the SulfAtlas database Version 2.3.1 (Barbeyron et al., 2016).^1^ Reads per kilobase per million (RPKM = 1,000,000 × (number of reads mapped/gene length in kilobase pairs)/number of reads in sample) values were calculated to estimate the normalized relative abundance of individual gene families. The phylogenetic affiliation of CAZyme was determined using the lowest common ancestor algorithm adapted from the DIAMOND blast by searching against the nonredundant database (Buchfink et al., 2015; Zhao et al., 2020). There was no bias toward better predictions in individual taxonomic groups based on the CAZy database. However, the possibility of a systematic under-sampling of CAZyme diversity for individual clades cannot be excluded. The functional potentials annotations at the CAZyme family level were further grouped and assigned to substrate targets according to the common designations from the CAZyme database.^2^ Annotation of carbohydrate transporters was done using Kofam KOALA v1.3.0 against the KEGG database^3^ with the e-value cutoff of 10^−5^(Aramaki et al., 2020), and additionally checked by TCDB database (The Transporter Classification Database) (Saier et al., 2021).^4^ We used SignalP 6.0 (Teufel et al., 2022) and Deep TMHMM (Hallgren et al., 2022) to predict the secreted CAZymes and subcellular localization.

### 2.4. Glycan niches measures

CAZymes microbial diversity and glycan niche width were indicated by the Shannon index and Levins index, respectively (Peterson et al., 2011; Baltar et al., 2021). This article focused on the degradation of carbohydrates; therefore, glycan niches measures and all analyses in the results section were based on the degradative CAZymes classes (Aas, Ces, CBMs, PLs, and GHs) excluding GTs.

Levins index can be calculated using the following equations:


$$B=\frac{1}{\sum p_{j}^{2}}$$

Where *B* is glycan niche width indicated by Levins index, 𝑗 refers to individual CAZyme family genes, *p_j_* is the proportion of *j* abundance in the total CAZymes gene abundance of the taxa. To standardize Levins niche width to a scale of 0–1, we use the equation:


$$B_{A}=\frac{B-1}{n-1}$$

Where *B_A_* is the standardized Levins niche width, *n* is the total number of CAZymes families predicted in metagenomic fragments.

The Shannon-Wiener index was calculated by the following equation:


$$B_{i}=-\overset{n}{\underset{j=1}{\sum }}\left(p_{i,j}\ln p_{i,j}\right)$$

Where 𝐵_𝑖_ is the Shannon-Wiener index of CAZyme gene abundance evenness for bacterial taxon *i*; 𝑗 refers to individual CAZyme family genes, n is the total numbers of CAZymes families in taxa *i*, n*_i,j_* is the CAZymes family gene *j* abundance of the bacteria taxa *i*, *N_𝑖_* is the abundance of CAZyme genes in bacterial taxon 𝑖 excluding GTs, *p_i,j_* is the proportion of CAZymes family gene *j* abundance to *N*_𝑖_ of the bacteria taxa *i* (*j* = 1,2,3,4…*n*).

The overlapping index of glycan niche between taxa *i* and *k* (*O_i,k_*) was calculated using the Morisita-Horn index by the following equations (Peterson et al., 2011):


$$O_{i,k}=\frac{2\sum_{i}^{n}p_{i,j}p_{i,k}}{\sum_{i}^{n}p_{i,j}{}^{2}+\sum_{i}^{n}p_{i,k}{}^{2}}$$

Where *p_i,j_* is the proportion of CAZymes *i* in the total CAZymes abundance in taxa *j*; *p_i,k_* is the proportion CAZymes *i* in the total CAZymes abundance in taxa *k; n* is the total number of CAZymes families (*i* is 1, 2, 3, …*n*).

### 2.5. Statistical analysis

Principal Co-ordinates Analysis (PCoA) of metagenomic CAZymes data was done between free-living and particle-associated fractions in water and between the water column and sediment samples using Permutational Multivariate Analysis of Variance (PerMANOVA) (999 permutations) using the R package vegan (Oksanen et al., 2022). STAMP (Statistical Analysis of Metagenomic Profiles) was used to search for CAZymes families differences across the different groups between surface and bottom water and between free-living and particle-associated fractions (Parks and Beiko, 2015). Nonparametric statistics (Wilcoxon–Mann–Whitney test) were done to compare differences between the metagenomic data, and statistical significance was accepted for *p* < 0.05. Average values are given by the statistical mean and its standard deviation (SD). Spaa (Zhang and Ma, 2013) and ggplot2 (Ginestet, 2011) were used for ordination, diversity and niche calculation, and visualization, respectively.

## 3. Results

### 3.1. Environmental parameters and microbial composition

Environmental data for water and surface sediment in the inner shelf of the PRE are given in Supplementary Tables S1, S2. Salinity was over 32 throughout the water column, indicating the strong influence of offshore oceanic water in winter. The total dissolved inorganic nitrogen and phosphate concentrations were lower than 10 and 0.2 μmol L^−1^, respectively, yielding N/P far above the Redfield ratio (16:1), thus indicating potential phosphate limitation in the study region. Phosphate deficiency likely reduced Chl*a* concentration (0.74–1.53 μg L^−1^). These results indicate relative nutrient-poor status during the sampling time. The concentration of acid-volatile sulfide was seven times higher at St DS02 than at St MC01, likely related to the pollution by aquaculture since DS02 was close to artificial reefs around Wan shan Island.

**Figure 2:**
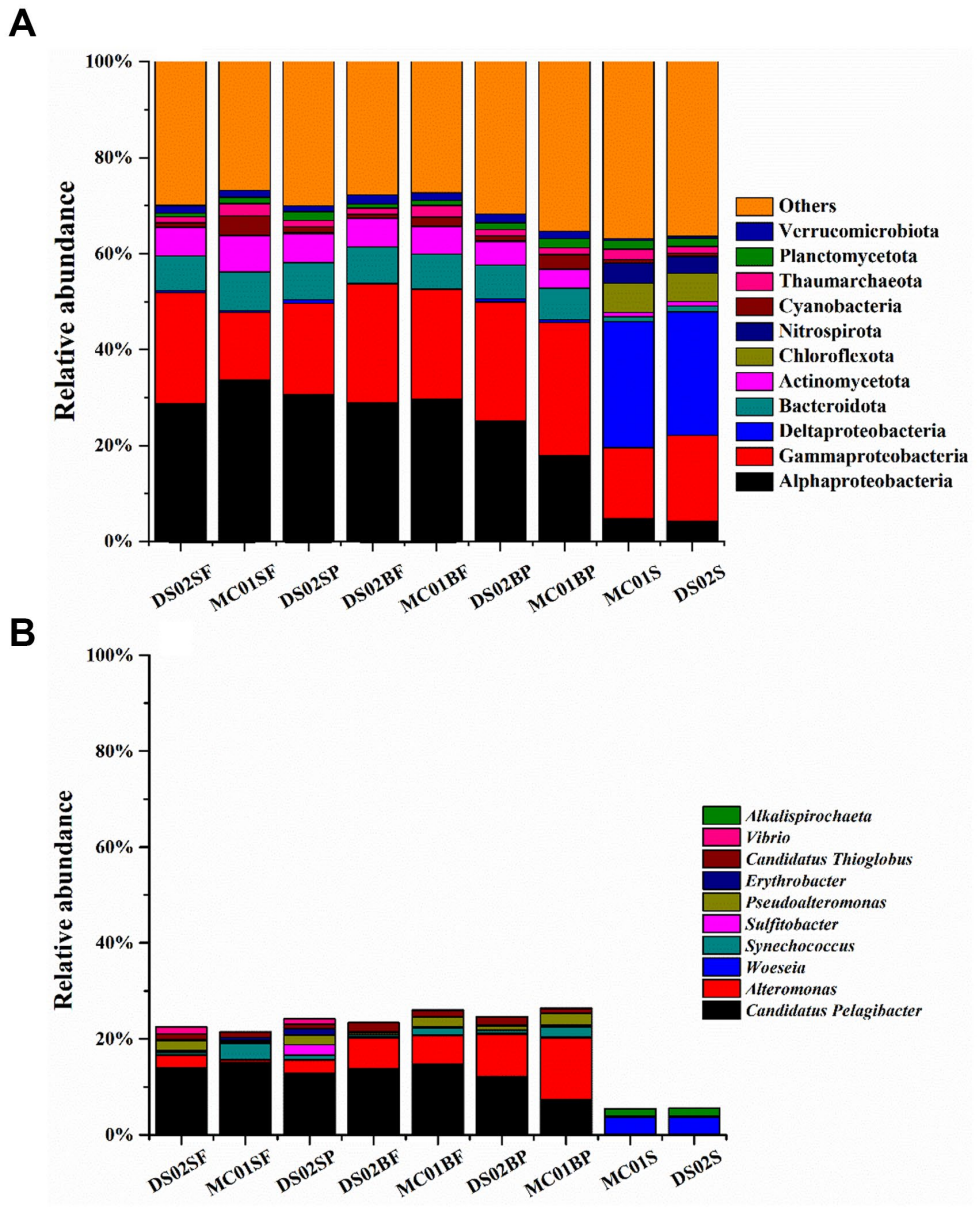
Bacteria community in phyla **(A)** and the top 10 genera of bacteria **(B)** in the inner shelf of the PRE (SF, surface free-living; SP, surface particles; BF, bottom free-living; BP, bottom particles; S, sediment).

The proportions of bacteria in the total microbial sequences were 72–82% and 84.6–86% in water and sediment, respectively (Supplementary Table S4). Bacterial communities showed significant differences between water and sediment (Figure 2A). Αlphaproteobacteria (17.99–33.63%), Gammaproteobacteria (14.18–24.82%), Bacteroidota (6.59–8.06%), Actinomycetota (3.86–7.51%) and Verrucomicrobiota (1.27–1.94%) were the most abundant phyla in the water. In the sediments, however, the contributions of Αlphaproteobacteria, Gammaproteobacteria, Bacteroidota, Actinomycetota, and Verrucomicrobiota were reduced, and Deltaproteobacteria (25.71–26.26%) were the most abundant. In addition, contributions of Chloroflexota (5.90–6.15%) and Nitrospirota (3.55–4.12%) in sediments were significantly higher than those in the water column in this study. At the genus level, the most abundant bacterial genera in this study were *Candidatus Pelagibacter* (7.33–14.89%), *Alteromonas* (0.52–12.95%) in water, and *Woeseia* (3.56–3.64%) in sediment, respectively (Figure 2B). A high proportion of *Alteromonas* in the bottom water particle samples (8.85–12.94%) was observed compared to all the surface water samples (0.52–2.71%).

### 3.2. Vertical and size-fractionated distributions of CAZymes families genes

The most abundant enzyme class genes were glycosyltransferases(GTs) (Supplementary Figure S1), followed by glycoside hydrolases (GHs). CEs **(**carbohydrate esterases), AAs (auxiliary activities associated with polysaccharide and lignin degradation), CBMs (carbohydrate-binding modules), and PLs (polysaccharide lyases). The relative abundance of genes encoding CAZymes classes differed significantly between water and sediment (Supplementary Figure S1), i.e., AAs, CEs, and PLs classes were significantly more abundant in the water samples (*p* < 0.05). This article focused on the degradation of carbohydrates, therefore, this study showed the annotation results on the degradative CAZymes genes except for GTs in the following sections. The CAZymes gene frequency of Σ (GHs + CBMs + CEs + PLs) was 0.55% in water and 0.36% in sediment, respectively (Supplementary Table S5). In this study, a total of 279 CAZymes families were identified, including 119 GHs and 79 GTs, 35 CBMs, 23 PLs, 13CEs, and 10 AAs (Supplementary Table S6, the top GH, CE, AA, PL, and CBM families were shown in Supplementary Figures S2–S4, and their main functions were listed in Supplementary Table S7).

**Figure 3:**
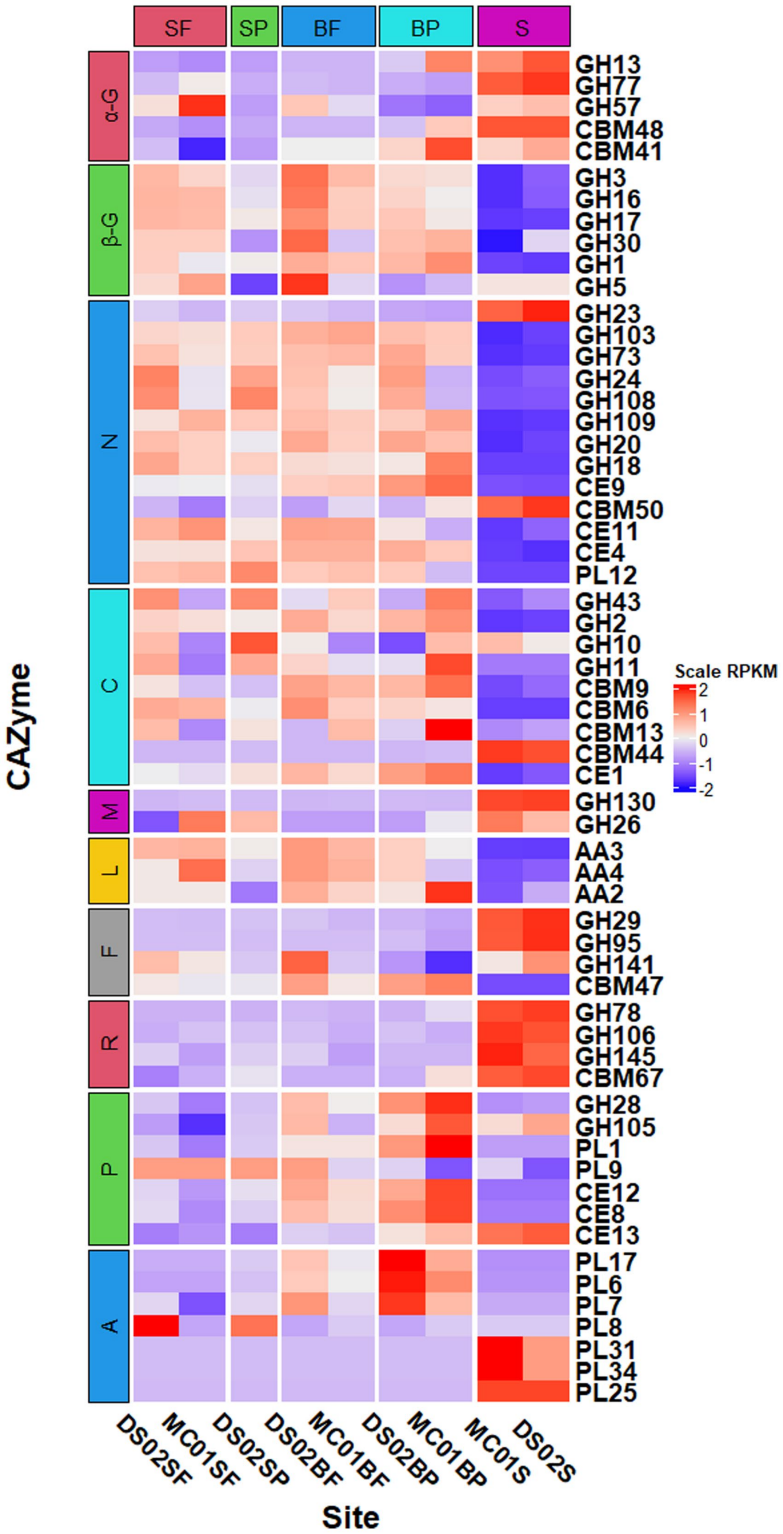
Heatmap of normalized RPKM of CAZyme families in different samples (SF, surface free-living; SP, surface particles; BF, bottom free-living; BP, bottom particles; S, sediment). The specified functional categories of CAZymes are shown: (1) α-G, targeting α-linked glucan; (2) β-G, targeting β-linked glucan; (3) N, targeting nitrogen-containing polysaccharides; (4) C, targeting cellulose and hemicellulose; (5) M, β-mannosides; (6) L, targeting lignin or lignocellulose by oxidative pathway; (7) F, targeting fucose containing polysaccharides; (8) R, targeting rhamnose containing polysaccharides; (9) P, targeting pectin or pectate; (10) A, targeting alginate/alginic acid.

**Figure 4:**
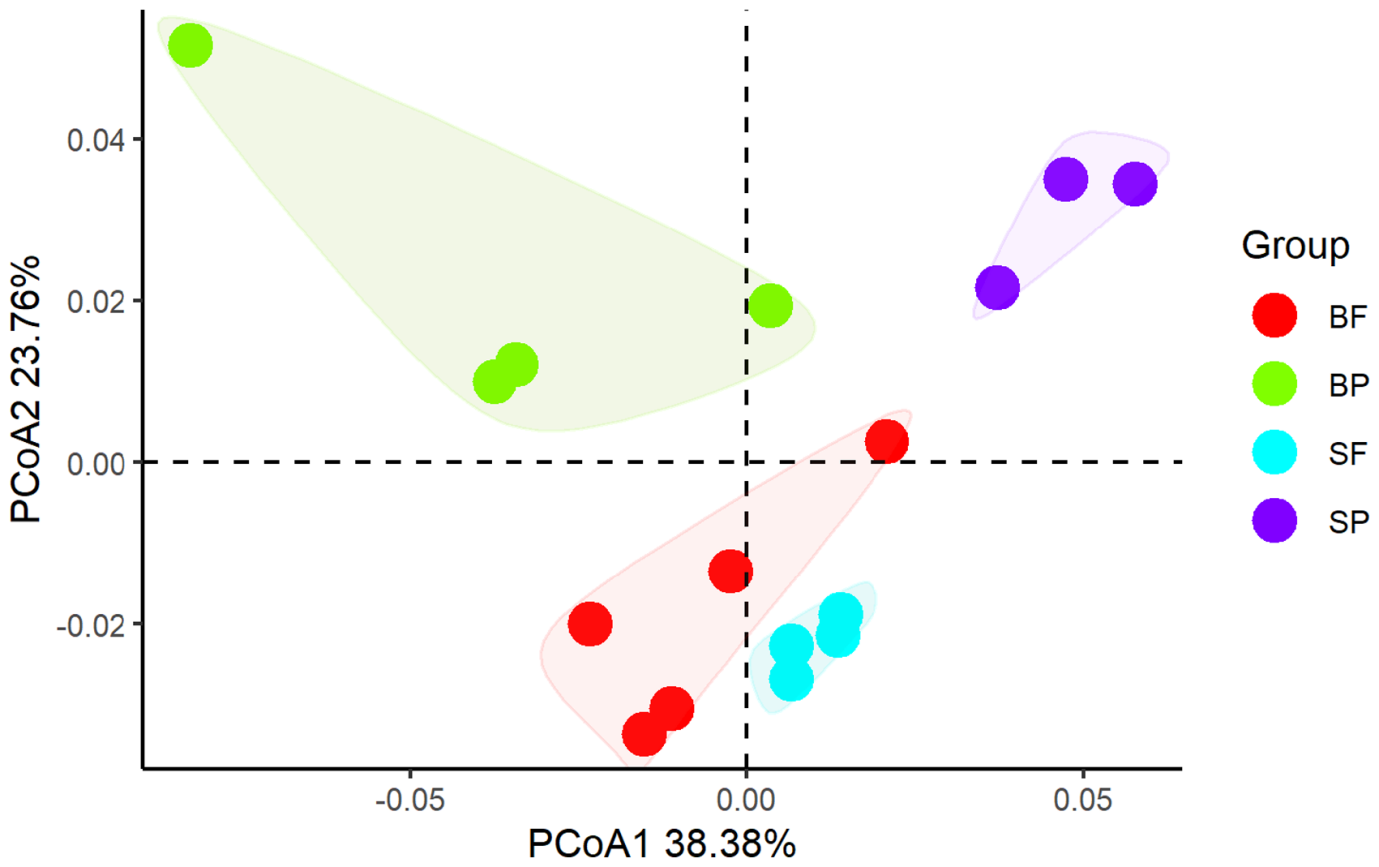
Principal coordinate analysis of CAZymes compositions between free-living bacterial fraction and particle-associated bacterial fraction in surface and sediment (SF, surface free-living; SP, surface particles; BF, bottom free-living; BP, bottom particles; S, sediment).

Principal coordinate analysis of CAZymes genes (Figure 3) and heat maps (Figure 4) indicate the presence of different CAZymes gene compositions between free-living bacterial fraction and particle-associated bacterial fraction (PERMANOVA, *R*^2^ = 0.59, *p* < 0.05) (Figure 3) and between water and sediment (PERMANOVA, *R*^2^ = 0.20, *p* < 0.001). The FL fraction has more abundant genes encoding AA3, 4, 6 (for lignin-cellulose), GH16, GH17, GH30 in surface (laminarinase), GH3 and GH5 (β-glucanase or glucosidase for oligo β-glucan), GH31, GH57, GH4, CBM6, CE3, CE11 than PA (Wilcoxon–Mann–Whitney test, p < 0.05) (Supplementary Figures S2, S3). In addition, the relative abundance of PLs genes was significantly higher in the particle-associated fraction of bottom waters (Wilcoxon–Mann–Whitney test, *p* < 0.05), such as PL1, 6, 7, and 17 (Figure 4). It reflected that the spectrum of PL genes was wider in the bottom particles and carboxyl acid polysaccharides were undergone deeply degraded by bacteria in the bottom water. The relative gene abundance of CBM48, 50, 67, and 44 was higher in sediment than in water (Wilcoxon–Mann–Whitney test, *p* < 0.05) (Supplementary Figure S3), and associated with a higher abundance of their binding GHs genes in sediment, such as GH13, 77, and 31 targeting α-glucan, GH23 targeting peptidoglycan and GH78 (rhamnosidases).

### 3.3. Five classes of CAZymes distributions in bacterial communities

**Figure 5:**
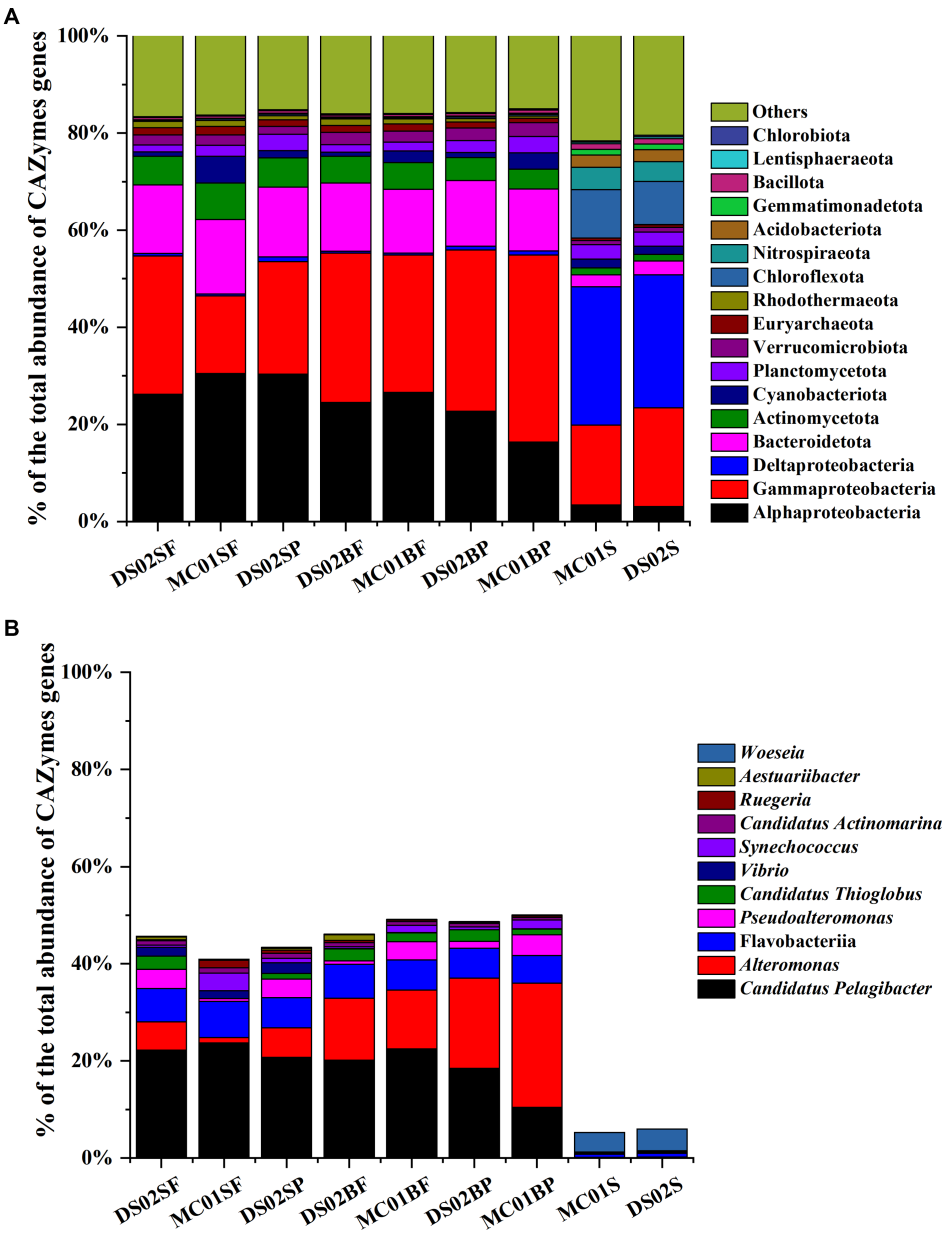
Contributions of the dominant taxa to the total CAZymes abundance (**A**: phylum; **B**: genus and class; SF, surface free-living; SP, surface particles; BF, bottom free-living; BP, bottom particles; S, sediment).

**Figure 6:**
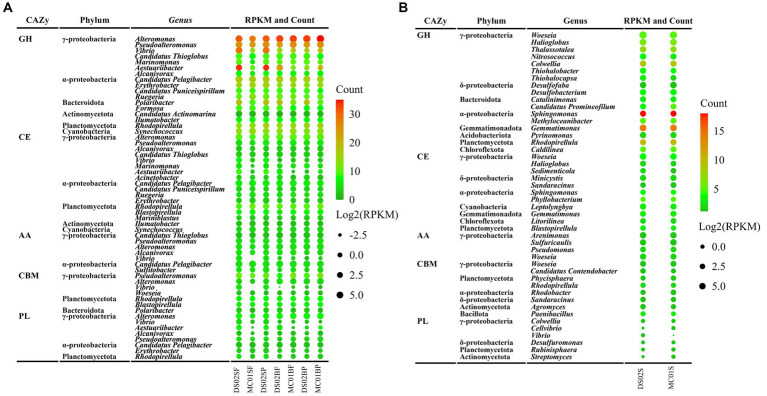
Distribution of the CAZymes within the major genera in the water **(A)** and in the sediment **(B)** in the inner shelf of the PRE (The dot color represents the CAZyme family numbers(Count) of the five CAZymes classes (GH, CE, AA, CBM, PL) in specific genera; The dot size indicates the abundance of five CAZymes classes (unit, RPKM) in specific genera; SF, surface free-living; SP, surface particles; BF, bottom free-living; BP, bottom particles; S, sediment).

Regarding the contributions of bacterial taxa to the total CAZymes genes, a similar distribution was observed compared with the bacteria composition at the phylum level (Figure 5A). At the genus level, for *Alteromonas* and *Candidatus Pelagibacter,* their contributions to the total CAZymes genes abundance ranged from 1.1–25.5% and 10.5–23.7% in water (Figure 5B), respectively. Figure 6A shows the counts of the number and relative abundance of GHs, PLs, CEs, AAs, and CBMs genes at the genus level. For GHs, the top contribution genera for GHs were i) *Alteromonas*, *Pseudoalteromonas*, *Vibrio*, *Candidatus Thioglobus*, *Aestuariibacter*, *Marinomonas* (Gammaproteobacteria), ii) *Candidatus Pelagibacter*, *Erythrobacter,* and *Ruegeria* (Alphaproteobacteria), iii) *Polaribacter* and *Formosa* (Bacteroidota), and iv) *Rhodopirellula* (Planctomycetota genus) (Figure 6A). PLs gene distribution in bacteria was similar to GHs, in which genus *Alteromonas* had the highest abundance and number of PLs genes in water. *Candidatus Pelagibacter* were the most prominent in CEs and AAs genes in water (Figure 6A). In addition, AAs genes were characterized by high abundance in Roseobacter Clade (*Sulfitobacter, Roseovarius, Ruegeria, Marinovum, Planktomarina*) belonging to Alphaproteobacteria. AAs are fundamental to facilitating lignin oxidation, even though they can not degrade lignin directly. Therefore, the wide distribution of genes encoding AAs in Alphaproteobacteria indicated that they were primary candidates to assist lignin-modifying enzymes for lignin degradation in this region. Unlike the distribution of CEs and AAs, no CBMs family genes were detected in *Candidatus Pelagibacter,* although they contained abundant CAZymes genes (Figure 6A). In contrast, besides *Pseudoalteromonas, Vibrio, Alteromonas* (Gammaproteobacteria) and *Polaribacter* (Bacteroidota), *Rhodopirellula, Mariniblastus, Blastopirellula, Planctomyces, Schlesneria, Gemmata* (Planctomycetota) and *Pedosphaera, Roseibacillus, Prosthecobacter* (Verrucomicrobiota) were rich in CBMs genes in water, suggesting these communities were specialists for complex polysaccharide degradation.

In this study, *Woeseia* (Gammaproteobacteria) was the dominant genus in sediment concerning the relative abundance of CAZymes genes (Figure 6B). However, regarding the numbers of CAZyems families, *Sphingomonas* (Alphaproteobacteria), *Gemmatimonas* (Gemmatimonadetes), *Colwellia and Halioglobus* (Gammaproteobacteria), *Rhodopirellula* and *Blastopirellula* (Planctomycetota) showed a high diversity of CAZymes in sediment (Figure 6B).

### 3.4. The glycan niche index in bacterial communities

**Figure 7:**
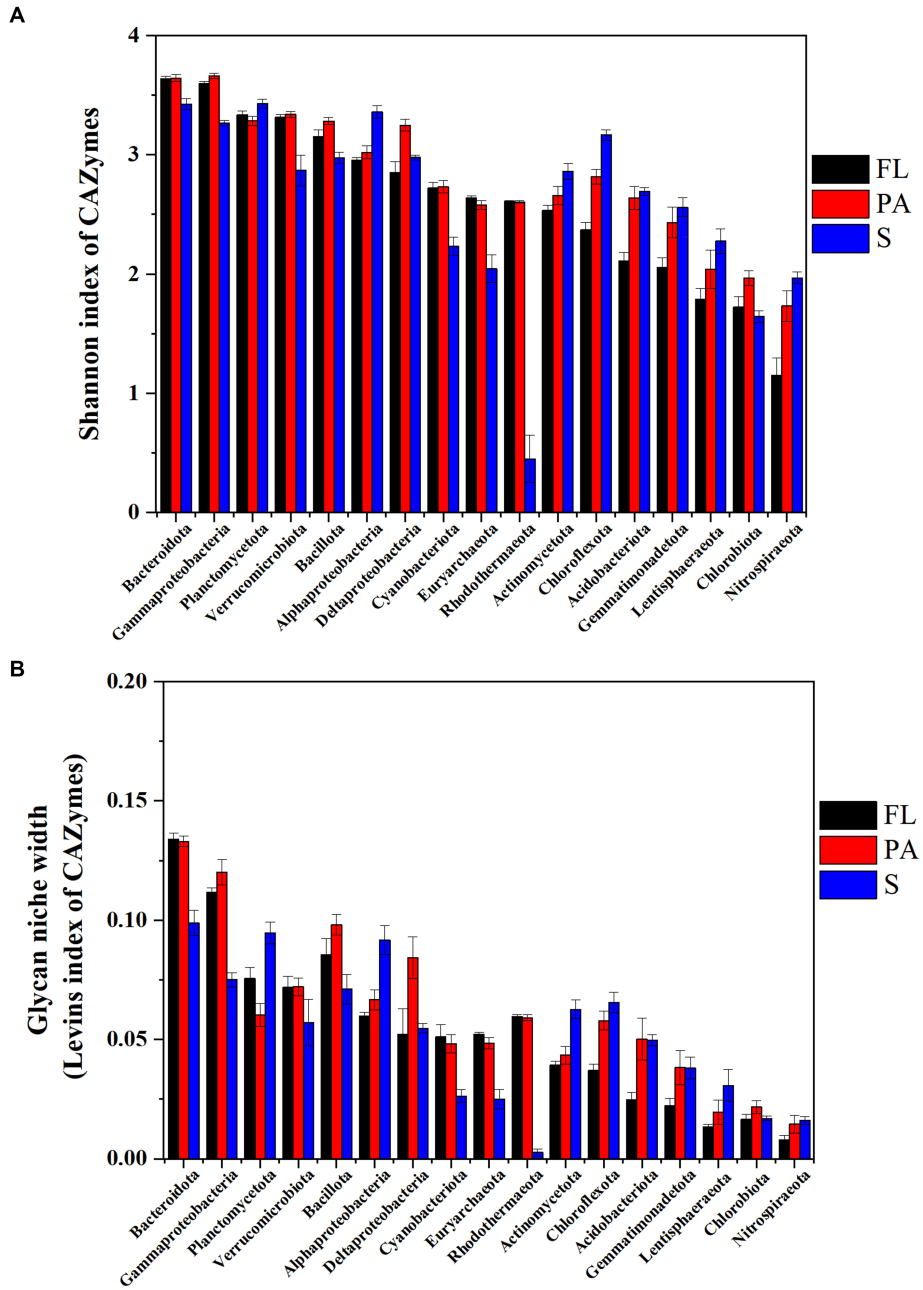
CAZymes diversity indicated by Shannon- index **(A)** and glycan niche width indicated by Levins index **(B)** (FL, free-living fraction; PA, particle-associated fraction; S, sediment).

In water, the Levins index and Shannon index generally showed a similar pattern of diversity of CAZymes families at the phylum level (Figure 7). The highest Shannon diversity index was found in Bacteroidota (3.64), Gammaproteobacteria (3.63), Planctomycetota (3.31), and Verrucomicrobiota (3.32) (Figure 7) in water, while in the sediment the index from high to low was found in Planctomycetota (3.43), Bacteroidota (3.43), Alphaproteobacteria (3.36), Gammaproteobacteria (3.27), and Chloroflexota (3.16), suggesting these communities had potentially wide glycan niches (Figure 7). At the genus level, the highest Levins index and Shannon index were observed in *Alteromonas* and *Pseudoalteromonas* in water and *Sphingomonas* in sediment, respectively (Supplementary Figure S5).

Taxa with a wider niche width usually have higher metabolic flexibility, thus being less affected by environmental fluctuations, whereas rare taxa were more easily influenced by environmental disturbances (e.g., the difference in substrate availability between FL and PA fractions). A higher glycan niche width in PA than in FL was seen for some relatively rare taxa (Chloroflexota, Nitrospiraeota, Lentisphaeraeota, Gemmatimonadetota, Bacillota, and Acidobacteriota) (Wilcoxon–Mann–Whitney test, *p*<0.05), but not for the abundant taxa (Figure 7). These rare taxa had more abundant CAZymes targeting algal cell wall-related compounds containing pectin, fucose, mannose, fucoidan and rhamnose, and other complex polysaccharides. Therefore, the high glycan niche for these relatively rare taxa in PA was related with that the estuary tends to have larger particles (i.e., larger phytoplankton and detritus). *O_i,k_* index between Planctomycetota and Verrucomicrobiota in water was over 0.8 with the highest in the PA fraction (0.93) (Table 1), suggesting the high degree overlap of glycan niche for these two phylum communities. In addition, a moderate degree overlap index *O_i,k_* (0.5–0.8) was found among Planctomycetota, Verrucomicrobiota, Bacteroidota, and Gammaproteobacteria. At the genus level, the high degree overlap index was found between *Alteromonas* and *Pseudoalteromonas* (the mean of 0.75) and between *Rhodopirellula* and *Mariniblastus* (the mean of 0.73) in all water samples.

**Table 1:** The overlap index (*O_i,k_*) of glycan niches at the phylum level.

Free-living fractions	P1	P2	P3	P4	P5	P6	P7	P8	P9	P10	P11	P12	P13	P14	P15	P16	P17
P1/Acidobacterota	0.00																
P2/Actinobacterota	0.10	0.00															
P3/Armatimonadetota	0.05	0.04	0.00														
P4/Bacteroidetota	0.17	0.23	0.12	0.00													
P5/Candidatus Bathyarchaeota	0.00	0.00	0.00	0.00	0.00												
P6/Candidatus Marinimicrobiota	0.10	0.18	0.09	0.48	0.00	0.00											
P7/Chloroflexota	0.03	0.15	0.01	0.29	0.00	0.38	0.00										
P8/Cyanobacteria	0.19	0.44	0.15	0.37	0.00	0.30	0.16	0.00									
P9/Euryarchaeota	0.13	0.32	0.03	0.46	0.00	0.28	0.28	0.41	0.00								
P10/Baccilota	0.07	0.36	0.06	0.43	0.00	0.34	0.18	0.50	0.46	0.00							
P11/Gemmatimonadetota	0.10	0.37	0.07	0.31	0.03	0.48	0.25	0.60	0.53	0.50	0.00						
P12/Lentisphaerota	0.04	0.02	0.01	0.30	0.00	0.74	0.29	0.02	0.02	0.08	0.05	0.00					
P13/Nitrospirota	0.08	0.38	0.07	0.25	0.00	0.38	0.05	0.62	0.25	0.44	0.80	0.03	0.00				
P14/Planctomycetota	0.14	0.16	0.17	0.54	0.00	0.15	0.09	0.24	0.21	0.21	0.14	0.05	0.22	0.00			
P15/Proteobacteria	0.11	0.24	0.05	0.56	0.00	0.26	0.47	0.49	0.42	0.40	0.31	0.07	0.26	0.27	0.00		
P16/Rhodothermaeota	0.12	0.20	0.07	0.68	0.00	0.43	0.37	0.16	0.57	0.21	0.24	0.26	0.11	0.55	0.40	0.00	
P17/Verrucomicrobiota	0.14	0.25	0.08	0.56	0.00	0.19	0.12	0.40	0.28	0.38	0.32	0.05	0.42	0.80*	0.35	0.50	0.00
Particle-associated fractions	P1	P2	P3	P4	P5	P6	P7	P8	P9	P10	P11	P12	P13	P14	P15	P16	P17
P1/Acidobacterota	0.00																
P2/Actinobacterota	0.24	0.00															
P3/Armatimonadetota	0.20	0.01	0.00														
P4/Bacteroidetota	0.41	0.26	0.09	0.00													
P5/Candidatus Bathyarchaeota	0.00	0.00	0.00	0.00	0.00												
P6/Candidatus Marinimicrobiota	0.25	0.23	0.02	0.46	0.00	0.00											
P7/Chloroflexota	0.17	0.20	0.01	0.44	0.00	0.61	0.00										
P8/Cyanobacteria	0.33	0.39	0.04	0.31	0.00	0.37	0.17	0.00									
P9/Euryarchaeota	0.28	0.33	0.01	0.39	0.00	0.24	0.30	0.30	0.00								
P10/Baccilota	0.26	0.28	0.01	0.39	0.00	0.21	0.25	0.35	0.34	0.00							
P11/Gemmatimonadetota	0.37	0.34	0.04	0.46	0.02	0.50	0.33	0.51	0.41	0.32	0.00						
P12/Lentisphaerota	0.05	0.04	0.02	0.32	0.00	0.70	0.55	0.04	0.03	0.06	0.14	0.00					
P13/Nitrospirota	0.21	0.34	0.02	0.24	0.00	0.50	0.14	0.54	0.22	0.22	0.62	0.03	0.00				
P14/Planctomycetota	0.19	0.19	0.06	0.55	0.00	0.15	0.10	0.19	0.15	0.18	0.33	0.11	0.27	0.00			
P15/Proteobacteria	0.44	0.26	0.02	0.58	0.00	0.31	0.34	0.45	0.32	0.47	0.38	0.10	0.24	0.24	0.00		
P16/Rhodothermaeota	0.31	0.19	0.03	0.67	0.00	0.37	0.44	0.13	0.52	0.15	0.38	0.31	0.08	0.53	0.38	0.00	
P17/Verrucomicrobiota	0.18	0.19	0.06	0.59	0.00	0.17	0.11	0.25	0.19	0.23	0.41	0.11	0.31	0.93*	0.29	0.50	0.00
Sediment	P1	P2	P3	P4	P5	P6	P7	P8	P9	P10	P11	P12	P13	P14	P15	P16	P17
P1/Acidobacterota	0.00																
P2/Actinobacterota	0.59	0.00															
P3/Armatimonadetota	0.14	0.10	0.00														
P4/Bacteroidetota	0.26	0.49	0.30	0.00													
P5/Candidatus Bathyarchaeota	0.12	0.20	0.07	0.09	0.00												
P6/Candidatus Marinimicrobiota	0.00	0.00	0.00	0.01	0.00	0.00											
P7/Chloroflexota	0.32	0.43	0.08	0.42	0.25	0.00	0.00										
P8/Cyanobacteria	0.16	0.30	0.01	0.29	0.08	0.00	0.53	0.00									
P9/Euryarchaeota	0.12	0.32	0.01	0.25	0.09	0.00	0.08	0.03	0.00								
P10/Baccilota	0.19	0.21	0.17	0.16	0.11	0.00	0.31	0.15	0.01	0.00							
P11/Gemmatimonadetota	0.48	0.42	0.06	0.38	0.06	0.00	0.64	0.70	0.03	0.22	0.00						
P12/Lentisphaerota	0.03	0.01	0.07	0.09	0.02	0.12	0.02	0.01	0.01	0.02	0.04	0.00					
P13/Nitrospirota	0.23	0.32	0.00	0.24	0.00	0.00	0.49	0.72	0.01	0.20	0.75	0.00	0.00				
P14/Planctomycetota	0.21	0.31	0.23	0.41	0.45	0.00	0.13	0.05	0.22	0.22	0.11	0.14	0.06	0.00			
P15/Proteobacteria	0.50	0.34	0.06	0.31	0.13	0.00	0.44	0.33	0.20	0.21	0.49	0.03	0.46	0.20	0.00		
P16/Rhodothermaeota	0.31	0.13	0.00	0.06	0.00	0.00	0.12	0.00	0.00	0.04	0.34	0.00	0.03	0.00	0.12	0.00	
P17/Verrucomicrobiota	0.18	0.15	0.29	0.39	0.01	0.00	0.12	0.08	0.16	0.11	0.15	0.15	0.04	0.34	0.15	0.05	0.00

Moderate degree of overlap, 0.5 < *O_i,k_* < 0.5–0.8; *high degree of overlap, *O_i,k_* > 0.8.

### 3.5. Niche difference of bacteria in CAZymes families-substrate specificities

#### 3.5.1. Nitrogen-containing polysaccharide-specific bacterial communities

**Figure 8:**
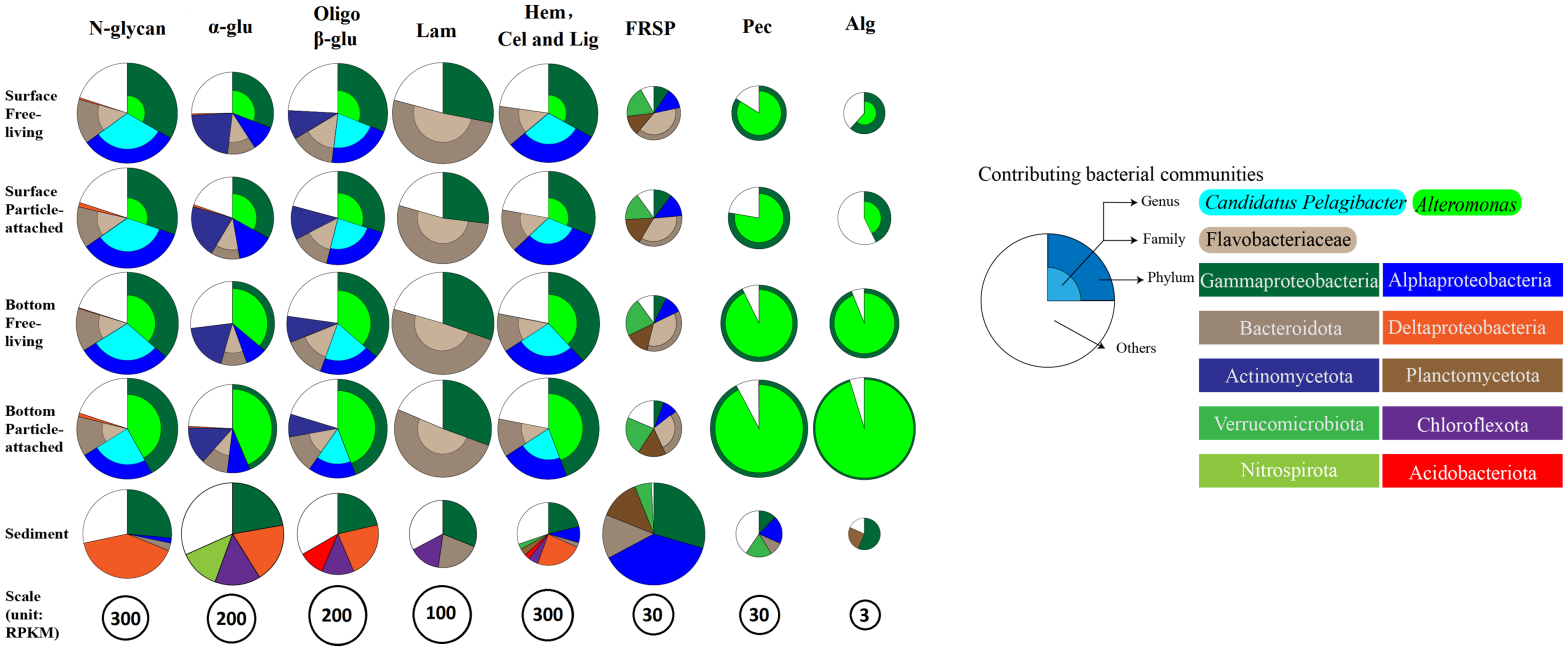
Glycan niches of dominant taxa in CAZymes families-substrate specificities in the inner shelf of the PRE (The specified functional categories of CAZymes are shown: (1) N-glycan, targeting nitrogen-containing polysaccharides; (2) α-glu, targeting α-linked glucan; (3) Oligo β-G, targeting oligo β-linked glucan; (4) Hem, Cel and Lig, targeting cellulose, hemicellulose and lignin; (5) FRSP, targeting sulfated fucose and rhamnose containing polysaccharides; (6) P, targeting pectin and pectate; (7) Alg, targeting alginate or alginic Acid).

**Figure 9:**
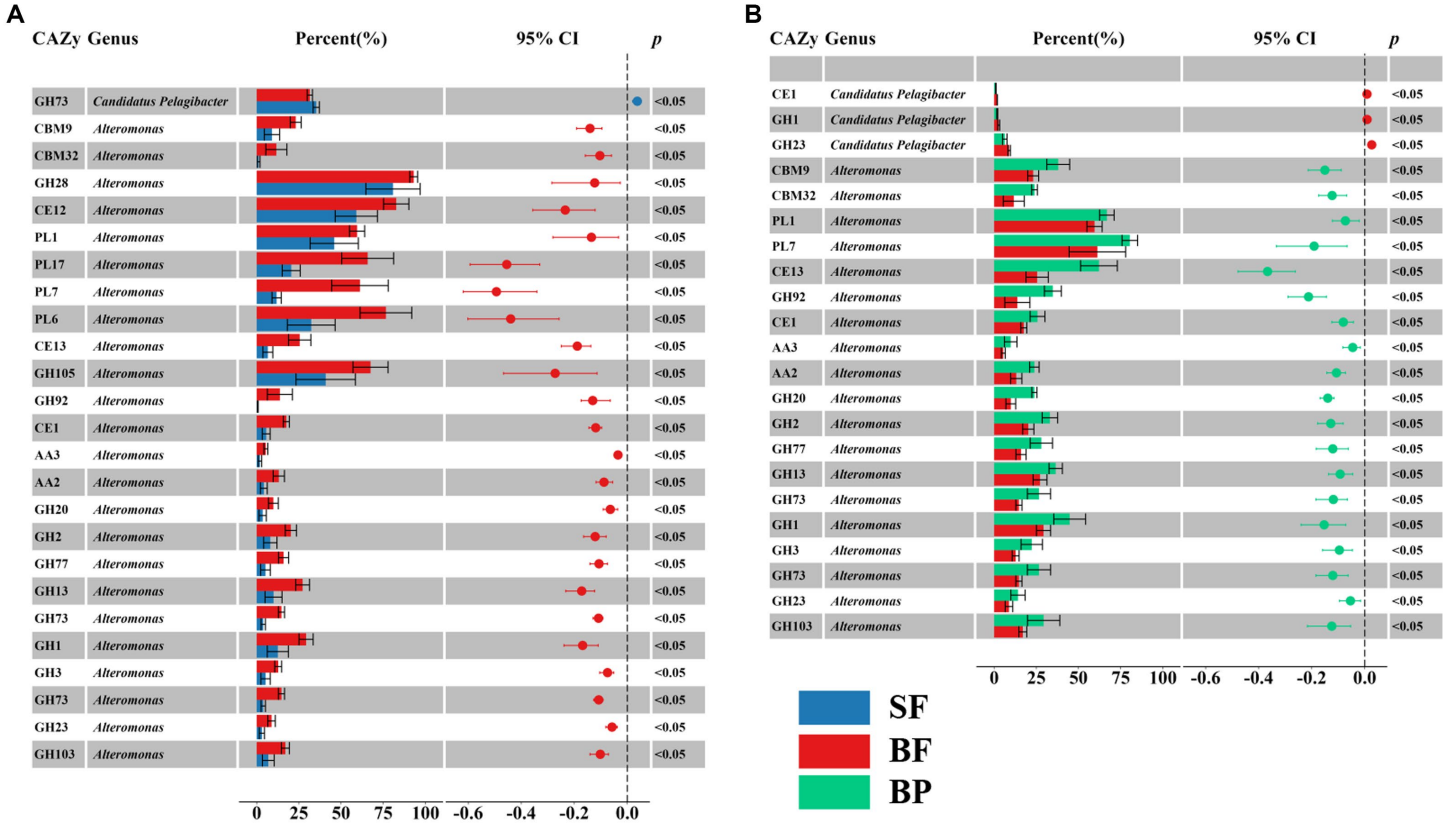
Significant difference in contributions of selective CAZymes abundance of *Alteromonas* and *Candidatus Pelagibacter* to the total CAZymes family abundance between surface free-living fraction (SF) and bottom free-living fraction (BF) (**A**), and between bottom particle-associated fraction and bottom free-living fraction (BF) (**B**).

The glycan niche separations were found for the dominant taxa as shown in Figure 8 according to CAZymes families-substrate specificities (Supplementary Table S8). The main N-glycan-specific phyla were Alpha- and Gammaproteobacteria and Bacteroidota in water and Delta- and Gammaproteobacteria in sediment at the phylum level, respectively. At the genus level, *Candidatus Pelagibacter* is primarily associated with enzyme families (GH103, GH23, 73, 108, 24, and PL12) that encode the degradation of nitrogen-containing polysaccharides such as peptidoglycan and other amino sugars (Supplementary Figure S6A), with the highest contribution found in the free-living cells of the surface waters for GH73 (Wilcoxon–Mann–Whitney test, *p* < 0.05) (Figure 9A). In contrast, *Alteromonas* dominated GH23, 103, and 73 in bottom waters rather than in surface waters (Wilcoxon–Mann–Whitney test, *p* < 0.05) (Figure 9A). With respect to the contributions of *Candidatus Pelagibacter* and *Alteromonas* to the total of GH23, 103, and 73 gene abundances, significant negative correlations were observed between these two genera for GH23 (*R*^2^ = 0.13), GH103 (*R*^2^ = 0.75) and GH73 (*R*^2^ = 0.88) in water (Supplementary Figure S7). These negative correlations suggest a competitive relationship and a pronounced niche separation between *Candidatus Pelagibacter* and *Alteromonas* in the metabolism of nitrogen-containing polysaccharides occurred in the coastal zone of the PRE.

GH109 is a family of N-acetylhexosaminidase (an exoglycosidase that catalyzes the hydrolysis of terminal, non-reducing β-*N*-Acetylgalactosamine and glucosamine residues from oligosaccharides) targeting chondroitin sulfate, oligo-chitin, muramic acid, N-glycans attached to proteins and the other glycoconjugates. In addition, GH33 is acetylneuraminyl hydrolase with the hydrolysis function of glycoconjugates. Interestingly, the GH109 and GH33 family gene was highly enriched in anaerobic Planctomycetota, Bacteroidetes, and Verrucomicrobiota (Supplementary Figure S8). GH18 (chitinases) and GH20 (β-*N*-acetylglucosaminidase) are involved in the degradation of chitins, and AA10 (lytic polysaccharide monooxygenases, LPMOs) oxidize the crystalline chitin to produce chitooligosaccharides (Jiang et al., 2022). *Pseudoalteromonas* and *Vibrio* were the primary candidates for GH18, GH20 (Supplementary Figure S6A), and AA10 in this study. In the inner shelf of the PRE, 30.6 ± 8.9% of AA10 was assigned to *Pseudoalteromonas* in the particle-attached fraction suggesting the importance of Gammaproteobacteria *Pseudoalteromonas* in the initial degradation of crystalline chitin by oxidative pathway.

#### 3.5.2. Storage polysaccharide-specific bacterial communities

With respect to β-glucans, Bacteroidota and Gammaproteobacteria were the prominent phyla with laminarin (β-1, 3-glucans, the main storage polysaccharides in algae) degrading enzymes (Figure 8). Enzymes from different enzyme families process the laminarin degradation pathway. Endo-glucanases (GH16 and GH17) often cleave the backbone of laminarin (β-1,3-linked glucose main chain), and GH30 enzymes remove the β-1,6 side chain glucose. The obtained oligosaccharides are subsequently hydrolyzed into glucose by GH3, 1 and 5 enzymes, and other glucosidases. In water, the dominant genera containing GH16 enzyme genes were *Lewinella*, *haeodactylibacter*, and *Polaribacter* (Bacteroidota) (Supplementary Figure S6B). For GH17 and GH30, *Formosa,* (a member of the family Flavobacteriaceae), was the dominant genus encoding for these two enzymes in water (Supplementary Figure S6B). The overwhelmingly dominant bacteria for oligo-β-glucan degradation (GH1 and GH3) were *Alteromonas* and *Candidatus Pelagibacter* in water (Supplementary Figure S6B). In addition, *Alteromonas* showed a significantly higher level of GH3 (25.3%) and GH1 (48.99%) in particle-attached bacteria of bottom waters than those in free-living fraction (Wilcoxon–Mann–Whitney test, *p* < 0.05) (Figure 9B). Besides Proteobacteria and Bacteroidota, the main storage polysaccharide-specific bacterial phyla were Actinomycetota (for α-glucan) in water and Chloroflexota (for α-glucan and β-glucan) and Nitrospirota (for α-glucan) in sediments, respectively. The CAZymes families GH13, 77, 15, 37, 97, 65, and CBM48 are involved in the degradation of α-linkages in glucan (e.g., starch and pullulan). At the genus level, *Alteromonas* dominated GH13 and 77 genes in water, specifically in the particle-attached bacterial fraction in bottom waters (Figure 9B), for example, 40.8% at MC01BP and 35.1% at DS02BP with respect to GH13. In the sediment, the dominant bacterial genus for degradation of α- and β- linked glucan were *Woeseia* due to their enrichment genes encoding GH13, GH77, CBM48, GH3, and GH 1 (Supplementary Figure S6B).

#### 3.5.3. Hemicelluloses, celluloses, and lignin-specific bacterial communities

GH43 (β-xylosidase; α-L-arabinofuranosidase), GH2 (arabinosidases), GH5 (endo-β-1, 4-glucanase / cellulase; endo-β-1, 4-xylanase) are responsible for degradation of hemicelluloses and celluloses. CE1 targets a large variety of substrates including the deacetylation of xylan. Most GH43 was assigned to *Pseudoalteromonas*, and GH2 and CE1 were assigned to *Alteromonas*. *Candidatus Pelagibacter* dominated GH5 targeted for oligo-glucan (Supplementary Figure S6C). For lignin degradation, *Candidatus Pelagibacter* and *Alteromonas* were dominating the AA3 family (Supplementary Figure S6C) and AA6 genes. AA3 belongs to the glucose-methanol-choline (GMC) oxidoreductases family involved in lignocellulose degradation together with other AA-enzymes such as peroxidases (AA2). AA6 can be involved in the production of extracellular oxyradicals for lignin modification via Fenton action and subsequent break of the lignin barrier to enhance the binding of CAZymes to lignocellulose. A high abundance of AA3, AA2, and GH2 families was found in *Alteromonas* only in bottom water samples, in particular in the particle-attached fraction (Wilcoxon–Mann–Whitney test, *p* < 0.05) (Figure 9B).

#### 3.5.4. Acidic-polysaccharides-specific bacterial communities

Notably, *Alteromonas* was proved to be the specialist for degradation of pectinaceous polysaccharides, with PL1 (pectate lyase, cleaving α-(1, 4)-linked D-galacturonan), GH28 (both endo and exo acting polygalacturonases), GH105 (unsaturated glucuronyl/galacturonyl hydrolases), CE8 (pectinesterase), CE12 (pectin acetylesterase) predominating (Supplementary Figure S6D). Furthermore, PL6, 7, and 17, which contain members catalyzing the depolymerization of alginate, were also dominated by *Alteromonas*. For example, the contribution of genes from *Alteromonas* to the gene abundance of PL6 were 32.34 ± 13.17%, 26.71 ± 14.15%, 76.59 ± 15.32%, 91.76 ± 2.61% in SF (surface free-living fraction), SP (surface particle-associated fraction), BF (bottom free-living fraction), and BP (bottom particle-associated fraction), respectively (Supplementary Figure S6D).

Our results also showed that Flavobacteriales, Gammaproteobacteria, Planctomycetota, and Verrucomicrobiota played an important role in the debranching of fucose- and rhamnose-rich sulfated heteropolysaccharides (FRSP) in the coastal PRE (Figure 8). For the genes encoding the fucosidase GH29 family, the metagenomic results showed that *Polaribacter* (a member of the family Flavobacteriaceae) and *Halioglobus* (members of the class Gammaproteobacteria) were the dominant genera, accounting for up to 12.17 and 9.24% of the total GH29 gene abundance in water, respectively (Supplementary Figure S6E). CBM47 (Fucose-binding activity) gene was rich in the Planctomycetota genera *Planctomyces* (12.94%) and *Rhodopirellula* (6.64%) in water. Furthermore, multiple sulfatase genes were detected in Flavobacteriales, Planctomycetales, and Verrucomicrobiales (Supplementary Figure S9). Regarding rhamnose-rich heteropolysaccharides, the GH78 family (rhamnosidase) genes were significantly less abundant than the GH29 family genes in the metagenomes. *Candidatus Pelagibacter* were dominating the genes encoding the GH78 family except for samples of MC01BP and *Rhodopirellula* were dominating CBM67 (binding rhamnose containing polysaccharide) in water, respectively. In the sediment, most GH29 genes were assigned to the *Halioglobus*, *Pseudoalteromonas* (Gammaproteobacteria), and *Colwellia* (Flavobacterium). The contributions of *Rhodopirellula* (Planctomycetota) to GH78 gene abundance were the highest with 16.60% at genus level in sediment (Supplementary Figure S6E).

### 3.6. Polymeric carbohydrate utilization mechanisms in different bacterial communities

In this study, the most dominant bacterial taxa involved in polysaccharide degradation were *Alteromonas*, *Candidatus Pelagibacter,* and the family Flavobacteriaceae. To better understand their carbohydrate uptake strategies, we additionally analyzed the carbohydrate transportation system in *Alteromonas*, *Candidatus Pelagibacter,* and Flavobacteriaceae. Generally, microbial transportation systems mainly include ATP-binding cassette (ABC) transporters (function for transportation of small molecular matter such as free amino acids and sugars), the phosphotransferase system (PTS), major facilitator super family (MFS) proton symporters, TonB-dependent receptors (TBDRs) and the starch utilization system C (SusC). The ‘selfish bacterium’ usually has genome encoding SusC transporters through which poly and oligo-saccharides come into the periplasm and then be hydrolyzed to monosaccharides for subsequent utilization with little loss of public products (oligosaccharides). In this study, SusC transporters and periplasmic protein TonB took most of the transportation system in Flavobacteriaceae (Table 2). Moreover, the presence of transporters (fucP, rhaP, and rhaQ, permease protein on fucose and rhamnose) (Table 2) and related CAZymes of the complex fucose- and rhamnose-containing polysaccharides in Flavobacteriaceae indicated that they were the specialists for consuming these polysaccharides in selfish mode, and were classed as moderate copiotrophs. This ‘selfish’ manner highly improves the payback of polysaccharide degradation enzymes. When substrates are sufficiently abundant, cells degrade polysaccharides to suitable sizes, providing extracellular hydrolysis products to the environment in sharing mode. Then, scavengers (beneficiaries) can directly take up the hydrolysis products created by the pioneers (Reintjes et al., 2020; Arnosti et al., 2021). In this study, the transporters in *Candidatus Pelagibacter* were all classified to the ABC transportation system, in which sugar transporters with low specificity took up the majority (Table 2). Moreover, the relative abundances (RPKM) of the transporter in *Candidatus Pelagibacter* are ten times as in *Alteromonas* and exhibited higher abundance in the free-living fractions than in the particle-attached bacteria (Table 2). Therefore, the scavenging mode by *Candidatus Pelagibacter* in free-living fractions through ABC transporters might be more prevalent and explain the dominance of *Candidatus Pelagibacter* for the assimilation of dissolved carbohydrates. Compared to *Candidatus Pelagibacter*, *Alteromonas* had a more complex transportation system, containing TonB, ABC, PTS, and MFS (Table 2). Due to the higher substrate specificity of TonB, PTS, and MFS than ABC (Lauro et al., 2009), *Alteromonas* may develop high efficiency in sugar transportation under copiotroph conditions, which enables *Alteromonas* to grow rapidly when nutrition is enough. This could explain the huge difference in the *Alteromonas* abundance between bottom and surface waters, particularly on bottom particles, where substrates are likely available in sufficiently dense patches (Ebrahimi et al., 2019). The most diverse CAZymes and sugar transportation systems found for *Alteromonas* on particles in this study were consistent with heterogeneous carbohydrate-defined niches, which was also found for phytoplankton blooms in the North Sea and the coast of the northern South China Sea (Dong et al., 2014; Francis et al., 2021). Therefore, differences in CAZymes, substrate binding, and importing mechanisms of these dominant bacterial communities indicate glycan niche speciation and contrasting strategies in carbohydrate uptake in the PRE.

**Table 2:** Abundance (unit, RPKM) of transporters for different structure polysaccharides in *Alteromonas*, *Candidatus Pelagibacter*, and Flavobacteriaceae.

Bacterial taxa	Transporter	KO_ID	SF	SP	BF	BP	*S*
g__*Alteromonas*	ABC.MS.S | multiple sugar transport system substrate-binding protein	K02027	0.97	1.34	5.78	9.87	0.00
	fucP | MFS transporter, FHS family, L-fucose permease	K02429	10.05	11.91	31.12	54.71	0.00
	lacY | MFS transporter, OHS family, lactose permease	K02532	0.29	0.39	2.21	2.61	0.00
	PTS-Glc-EIIA, crr | PTS system, sugar-specific IIA component	K02777	2.03	2.97	3.81	4.89	0.00
	tonB | periplasmic protein TonB	K03832	13.50	15.83	31.87	47.90	0.00
g_*_Candidatus Pelagibacter*	ABC.MS.P | multiple sugar transport system permease protein	K02025	36.58	32.45	36.30	28.25	0.08
	ABC.MS.P1 | multiple sugar transport system permease protein	K02026	64.17	52.43	65.68	47.22	0.00
	ABC.MS.S | multiple sugar transport system substrate-binding protein	K02027	128.22	73.63	128.79	91.25	0.08
	ABC.SS.A | simple sugar transport system ATP-binding protein	K02056	129.27	107.43	135.73	97.68	0.70
	ABC.SS.P | simple sugar transport system permease protein	K02057	153.16	107.41	159.41	116.30	0.09
	ABC.SS.S | simple sugar transport system substrate-binding protein	K02058	122.79	65.39	120.38	84.05	0.00
	aglK | alpha-glucoside transport system ATP-binding protein	K10235	1.18	0.68	0.69	0.60	0.00
	gtsA, glcE | glucose/mannose transport system substrate-binding protein	K17315	17.94	10.55	18.64	12.52	0.00
	gtsB, glcF | glucose/mannose transport system permease protein	K17316	4.70	3.54	4.85	3.50	0.00
	gtsC, glcG | glucose/mannose transport system permease protein	K17317	34.01	26.53	35.69	26.99	0.02
	lacK | lactose/L-arabinose transport system ATP-binding protein	K10191	5.11	4.42	5.62	4.95	0.00
	malK, mtlK, thuK | multiple sugar transport system ATP-binding protein	K10111	9.73	8.83	9.55	8.23	0.04
f_Flavobacteriaceae	ABC.MS.P1 | multiple sugar transport system permease protein	K02026	0.15	0.11	0.19	0.09	0.00
	fucP | MFS transporter, FHS family, L-fucose permease	K02429	5.05	5.66	5.19	3.88	1.29
	PTS-Fru2-EIIB | PTS system, fructose-specific IIB-like component	K11202	0.49	0.35	0.50	0.13	0.00
	PTS-Fru-EIIA, fruB | PTS system, fructose-specific IIA component	K02768	0.06	0.11	0.11	0.04	0.00
	PTS-Fru-EIIB, fruA | PTS system, fructose-specific IIB component	K02769	0.19	0.39	0.26	0.31	0.00
	rhaP | rhamnose transport system permease protein	K10560	0.37	0.15	0.37	0.21	0.00
	rhaQ | rhamnose transport system permease protein	K10561	0.29	0.18	0.27	0.27	0.00
	susC | TonB-dependent starch-binding outer membrane protein SusC	K21573	5.41	7.85	4.57	3.94	0.03
	tonB |periplasmic protein TonB	K03832	5.64	8.35	3.94	2.76	1.25
	xylE | MFS transporter, SP family, xylose:H+ symportor	K08138	0.82	2.02	0.54	0.97	0.41

## 4. Discussion

Relative nutrient-poor status was found in the inner shelf of the PRE in winter, which has been found in a previous study (Liu et al., 2017). Previous studies in oceans and soils have found that nutrient levels and the availability of nutrients (i.e., organic carbon and nitrogen) determine the relative abundances of specific metabolic genes (Louca et al., 2016; Gowda et al., 2022). Therefore, the CAZymes gene frequency of Σ (GHs + CBMs + CEs + PLs) during this sampling time was comparable to the non-bloom phase at Helgoland (North Sea) (0.4–0.7% during non-bloom vs. 1.1% during bloom phase) (Teeling et al., 2016), coinciding with the low level of Chl*a* concentration and poor nutrients status in this study.

In this study, CAZymes genes involved in the metabolism of nitrogen-containing polysaccharides contributed the largest proportions to the total abundance of GHs (∑(GH23, 103, 108, 24, 73, 18, and 20), 34.04% in water and 24.61% in sediment, respectively). Moreover, PRKM of genes encoding lysozyme (GH73, 103, 108, 109, 24) on the coast of the PRE showed 3–4 times higher than those samples in non-bloom water in the North Sea at station approximately 60 km offshore from the northern German coastline, while the PRKM of most CAZymes families genes in this study were comparable to those samples (Vidal-Melgosa et al., 2021). Notably, an enzyme that degrades peptidoglycan that is not secreted is probably related to cell wall biosynthesis or recycling, but one that is secreted might be related to degrading environmental peptidoglycan (Vollmer et al., 2008; Gilmore and Cava, 2022). In the secreted CAZymes pool, GHs gene abundance [∑(GH23, 103, 24, 73, 18, and 20)] also contributed the largest proportions to the total abundance of GHs, 36.27% in water and 53.30% in sediment, respectively (Supplementary Table S9). It was consistent with the results of a previous report that hydrolyses enzymes for degrading the polymer of N-actyl glucosamine dominated extracellular enzymatic activity at pristine offshore of PRE other than in eutrophic environments (Shi et al., 2019). Therefore, a high inventory of bacterial-derived necromass for microbial carbon turnover existed in the inner shelf of the PRE, due to a reduction in the supply of phytoplankton-derived organic matter in the coastal estuaries system in winter, coinciding with the low level of Chl*a* concentration during the sampling time.

The ɑ-diversity of CAZymes (Shannon index) in this study was lower than that of human gut microbes (Smits et al., 2017). Nevertheless, it was comparable to that of fungi in marine sediments (Baltar et al., 2021). In this study, the most abundant CAZymes and related genes and the widest glycan niche in the abundant bacterial taxa suggested their potential key roles in organic carbon utilization. *Alteromonas* were the most important genus according to both the glycan niche index and microbial abundance followed by *Candidatus Pelagibacter* in this study. *Alteromonas* accounted for up to 22.19% of the total CAZymes gene abundance in the bottom water, which is nearly twice their proportions to the total microbial abundance. In addition, the composition of CAZymes of *Alteromonas* revealed that they are not only metabolic generalists capable of utilizing a wide variety of organic compounds (e.g., peptidoglycan, oligo-β-glucan, α-glucan, cellulose, hemicellulose), but also are specialists for recycling pectin and alginate. Similar results have been reported in experiments and *in situ* field observations indicating *Alteromonas* spp. comprised high proportional actively growing bacterial population (Pedler et al., 2014).

In contrast, the glycan niche of *Candidatus Pelagibacter* was narrower than those of *Alteromonas*. For example, no CBM family genes were detected in *Candidatus Pelagibacter,* although they contained abundant CAZymes genes (Figure 6A). CBMs are the most prominent ancillary modules and serve as non-catalytic accessory modules that bind carbohydrates, thus enhancing the catalytic efficiency of the multi-modular CAZymes (Boraston et al., 2004). The absence of CBMs genes in *Candidatus Pelagibacter* confirmed their oligotrophic niches, as they prefer to use the small molecule(i.e., one-carbon metabolism)rather than complex high molecular weight polysaccharides (Malmstrom et al., 2005; Tripp et al., 2008; Sun et al., 2011; Carini et al., 2014). Nevertheless, a large abundance of ABC transporters genes (multiple sugar transport system substrate-binding protein and simple sugar transport system ATP-binding protein) matched to *Candidatus Pelagibacter*, supported that they take up a substantial mono sugar or oligosaccharide from primary production or PG fragments mediated by ABC transporter in the oceans (Giovannoni, 2017; Noell et al., 2021; Gilmore and Cava, 2022). In addition, our results were consistent with previous reports that they have more genes for the uptake of amino acids and other nitrogenous compounds than for the uptake of carbohydrates (Cottrell and Kirchman, 2000; Noell et al., 2021). These findings suggest that *Alteromonas* and *Candidatus Pelagibacter* were the key genera for a significant flux of DOC and nutrient mineralization at the surface and the bottom, respectively, in this study (McCarren et al., 2010; Sun et al., 2011).

Bacteroidota and Gammaproteobacteria harbored remarkably abundant CAZymes genes for degrading laminarin. In particular, Bacteroidota contributed to over 50% of these genes (Figure 8). Laminarin (a food storage polysaccharide in diatoms) is a major component in the ocean and plays a prominent ecological role and biogeochemical function in oceanic carbon export and energy flow from primary production to next trophic levels in the food web (Becker et al., 2020). In this study, the relative abundance genes encoding GH16, 17, 30 (laminarinase) in the surface PA fraction was significantly lower than those in the FL fraction (Figure 4). It implies that “particulate” laminarin was better prevented from enzymatic hydrolysis and dissolution within the intact diatom cells. In contrast, the dissolved laminarin released from diatom can be quickly hydrolyzed by extracellular laminarinase, but also by ‘selfish’ uptake by Bacteroidota cells with little diffusive loss (Reintjes et al., 2020). It also indicated that DOM quality plays a significant role in controlling the microbial community response (Liu S. T. et al., 2020; Kieft et al., 2021). Fucose- and rhamnose-rich anionic heteropolysaccharides, which are also found in TEP (Passow, 2002), can show extensive sulphation. Due to their complex structure, they are rather recalcitrant and are degraded more slowly (turnover time up to 1 month) than other polysaccharides, such as carbonylated polysaccharides α-1,4-galacturonan (turnover time of days to weeks) and β-1,3-glucan (turnover time of days) (Vidal-Melgosa et al., 2021). Previously, Planctomycetota were shown to colonize the surfaces of particles in the PRE, such as the diatom *Thalassiosira weissflogii* (Zhang et al., 2016; Ma et al., 2022). Thus, their enrichment of sulfatase (Supplementary Figure S9) and CBM genes in the surface water particle-attached fraction may be associated with the presence of fucose- and rhamnose-rich anionic sulfated heteropolysaccharides that are likely derived from phytoplankton in these samples. The results of this study were consistent with earlier findings on the degradation of anionic sulfated heteropolysaccharides by Flavobacteriales, Gamma-proteobacteria, Verrucomicrobiota and Planctomycetota (Wegner et al., 2013; Xing et al., 2015; Ma et al., 2022; Orellana et al., 2022). Furthermore, the CAZymes gene compositions revealed a close connection between microbiome and algae, which enable these communities to efficiently utilize carbohydrate carbon produced from algae (Gugi et al., 2015; Reintjes et al., 2019; Ma et al., 2022).

In this study, the vertical distribution reflected the difference in niches between layers and provided insights into carbon turnover and preservation. High abundance genes encoding α-glucosidases (GH13 and GH77) in sediment suggested that α-linked glucans (starch or pullulan) generally from algal, bacterial, and animal storage can be taken more rapid hydrolysis in sediment than in water. Similarly, rapid pullulan (α-linked glucans) degradation has also been found in all oxic and anoxic sediments from locations including the Baltic Sea, the eastern North Atlantic, and the Arctic Ocean (Arnosti, 2000). Furthermore, the significantly higher relative abundance of genes in sediment soil was found for fucosidases (GH 29 and 95), rhamnosidase (GH78, 106, and 145), and β-mannosides (GH130 and 26) involved in the utilization of cell wall polysaccharides (e.g., FRSP) compared to their abundances in water. These are enzymes for the hydrolysis of methyl pentose sugars, which are known as a potential carbon sink due to their challenge to degrading structure for bacteria compared to other monomers (Vidal-Melgosa et al., 2021). Generally, FRSP that diatoms secrete remains outside the cell or as part of the cell wall, and thus FRSP in POM also increases during the diatom bloom or post-bloom (Vidal-Melgosa et al., 2021). It has been suggested that increasing the concentration of secreted adhesive polysaccharides (TEP and their precursor) favors the aggregation of diatoms, accelerating their sinking velocity (Engel et al., 2004). This may explain the accumulation of FRSP in shallow sediments of the coast of the estuary during this study. The vertical distribution of GHs genes abundances further points to the selective degradation of polysaccharides with different bioavailability and that FRSP undergoes an extensive transformation in sediment rather than in water (Smith et al., 2019). Furthermore, RPKM of the PLs and GHs for acid polysaccharides were an order of magnitude below those of abundant GHs, CEs, and AAs targeting other substrates, indicating that acid polysaccharides might play a minor role in carbon turnover than nitrogen-containing polysaccharides, glucan, xylan, and lignin in the PRE inner shelf (Sun et al., 2012; Vidal-Melgosa et al., 2021).

With respect to the glycan overlap index, the high degree overlap of CAZymes was found between Planctomycetota and Verrucomicrobiota at the phylum level. At the genus level, the high degree overlap index was found between *Alteromonas* and *Pseudoalteromonas* and between *Rhodopirellula* and *Mariniblastus* in all water samples. These species pairs were close relatives based on phylogenetic analysis (Gauthier et al., 1995; Butler et al., 2007; Munoz et al., 2016). Therefore, carbohydrate preferences are more similar with increasing phylogenetic relatedness, since more closely related strains tend to have more similar metabolic profiles (Bryson et al., 2017; Fahimipour and Gross, 2020; Kieft et al., 2021). However, a higher overlap index means a competitive relationship for carbohydrate utilization. The paradox that species with similar glycan niches tend to co-occur can be explained by deterministic environmental filtering (Santillan and Wuertz, 2022). A specific environment that provides some set of resources will be occupied by species that demand these resources, whereas a different set of species will fit a different environment offering a different set of nutrients (Levy and Borenstein, 2013). The similar utilization of diatom-produced polysaccharides (i.e., sulfated polysaccharide) determined glycan niche overlap among these taxa (Planctomycetota, Bacteroidota, Verrucomicrobiota, Gammaproteobacteria). In this study, the abundance of *Alteromonas* was higher than those of *Pseudoalteromonas,* indicating low-level co-occurrence degree although they had similar CAZymes structures. It has been found that *Alteromonas* have a faster N uptake ability than *Pseudoalteromonas* (Arandia-Gorostidi et al., 2022), suggesting that N-poor status in this study might reduce the competitiveness of *Pseudoalteromonas*. Therefore, the deterministic processes played an important role in microbiome assembly (Teeling et al., 2016).

## 5. Conclusion

This study offers insight into the mechanisms of microbial communities to degrade polymeric carbohydrates in the inner shelf of the estuarine system. In this study, nutrients and Chl *a* indicated relative nutrient-poor status during the sampling time, inducing a high fraction of GHs genes targeting peptidoglycan (bacterial cell wall) and chitin. Furthermore, significant differences in CAZymes profiles were found between water and sediment and between free-living and particle-attached bacterial fractions in water. The vertical distribution of CAZymes genes suggested a selective degradation of polysaccharides with different bioavailability, and a higher degree of recalcitrant FRSP biotransformation in sediment rather than in water. Proteobacteria and Bacteroidota had the highest abundance and glycan niche width of CAZymes in water, respectively. Members of the Alphaproteobacteria (*Candidatus Pelagibacter*), Gammaproteobacteria (*Alteromonas, Pseudoalteromonas, and Woeseia*), and Flavobacteriaceae were among the most prominent responders to the polymer carbohydrates. At the genus level, the glycan niches width of the genus *Alteromonas* were the widest for degradation of laminarin, starch, and nitrogen-containing polysaccharides, carboxylic-acid polysaccharides, cellulose, and lignin, associated multiple sugar transporter in sharing modal, and marked with depth difference (BF > SF) for free-living fraction and size difference for bottom samples (BP > BF). While the functional potentials of *Candidatus Pelagibacter* showed dominance for substrates like oligo-glucan, nitrogen-containing polysaccharides, oligo-cellulose, and lignin-derived aromatic fragments. The most abundant CAZymes and related genes and the widest glycan niche in the abundant bacterial taxa suggested their potential key roles in organic carbon utilization. The high extent of glycan niche overlap for utilization of diatom-produced polysaccharides revealed a close connection between bacteria (e.g., Bacteroidota, Gammaproteobacteria, Planctomycetota, and Verrucomicrobiota) and algae. Collectively, CAZymes gene traits and their related transporters as a good proxy for assessing distinct ‘polymeric carbohydrate utilization types’ of the microbial communities indicated different bacterial strategies of polysaccharide degradation and the niches separation subject to polymeric carbohydrate substrates in the estuarine system.

## Data availability statement

The datasets presented in this study can be found in online repositories. The names of the repository/repositories and accession number(s) can be found in the article/Supplementary material.

## Author contributions

C-CS designed the manuscript and discussed the results and implications, and commented on the manuscript at all stages. W-JZ performed the annotation of the metagenome data and drew the figures, and did the sampling and chemical parameter analysis. Y-SW provided the research direction. W-ZY, HC, F-LS, Y-TW, M-LW, and AE helped to check the manuscript. All authors contributed to the article and approved the submitted version.

## Funding

This study was supported by the National Natural Science Foundation of China (Nos. 42073078 and U1901211), Independent Research Project of State Key Laboratory of Tropical Oceanography (LTOZZ2202), the Key Research and Development Program of Hainan Province (ZDYF2021XDNY131), and the Strategic Priority Research Program of the Chinese Academy of Sciences (No. XDA19060204).

## Conflict of interest

The authors declare that the research was conducted in the absence of any commercial or financial relationships that could be construed as a potential conflict of interest.

## Publisher’s note

All claims expressed in this article are solely those of the authors and do not necessarily represent those of their affiliated organizations, or those of the publisher, the editors and the reviewers. Any product that may be evaluated in this article, or claim that may be made by its manufacturer, is not guaranteed or endorsed by the publisher.

## Footnotes

^1^http://abims.sb-roscoff.fr/sulfatlas/index.html

^2^www.cazy.org

^3^https://www.genome.jp/kegg/

^4^https://tcdb.org/

## References

[ref1] Alderkamp A. C. Van Rijssel M. Bolhuis H. (2007). Characterization of marine bacteria and the activity of their enzyme systems involved in degradation of the algal storage glucan laminarin. FEMS Microbiol. Ecol. 59, 108–117. doi: 10.1111/j.1574-6941.2006.00219.x, PMID: 17233748

[ref2] Aramaki T. Blanc-Mathieu R. Endo H. Ohkubo K. Kanehisa M. Goto S.. (2020). KofamKOALA: KEGG Ortholog assignment based on profile HMM and adaptive score threshold. Bioinformatics 36, 2251–2252. doi: 10.1093/bioinformatics/btz859, PMID: 31742321 PMC7141845

[ref3] Arandia-Gorostidi N. Berthelot H. Calabrese F. Stryhanyuk H. Klawonn I. Iversen M.. (2022). Efficient carbon and nitrogen transfer from marine diatom aggregates to colonizing bacterial groups. Sci. Rep. 12:14949. doi: 10.1038/s41598-022-18915-0, PMID: 36056039 PMC9440002

[ref4] Arnosti C. (2000). Substrate specificity in polysaccharide hydrolysis: contrasts between bottom water and sediments. Limnol. Oceanogr. 45, 1112–1119. doi: 10.4319/lo.2000.45.5.1112

[ref5] Arnosti C. Wietz M. Brinkhoff T. Hehemann J. H. Probandt D. Zeugner L.. (2021). The biogeochemistry of marine polysaccharides: sources, inventories, and bacterial drivers of the carbohydrate cycle. Annu. Rev. Mar. Sci. 13, 81–108. doi: 10.1146/annurev-marine-032020-012810, PMID: 32726567

[ref6] Avci B. Kruger K. Fuchs B. M. Teeling H. Amann R. I. (2020). Polysaccharide niche partitioning of distinct Polaribacter clades during North Sea spring algal blooms. ISME J. 14, 1369–1383. doi: 10.1038/s41396-020-0601-y, PMID: 32071394 PMC7242417

[ref7] Baker B. J. Lazar C. S. Teske A. P. Dick G. J. (2015). Genomic resolution of linkages in carbon, nitrogen, and sulfur cycling among widespread estuary sediment bacteria. Microbiome 3:14. doi: 10.1186/s40168-015-0077-6, PMID: 25922666 PMC4411801

[ref8] Baltar F. Zhao Z. H. Herndl G. J. (2021). Potential and expression of carbohydrate utilization by marine fungi in the global ocean. Microbiome 9:106. doi: 10.1186/s40168-021-01063-4, PMID: 33975640 PMC8114511

[ref9] Bankevich A. Nurk S. Antipov D. Gurevich A. A. Dvorkin M. Kulikov A. S.. (2012). SPAdes: a new genome assembly algorithm and its applications to single-cell sequencing. J. Comput. Biol. 19, 455–477. doi: 10.1089/cmb.2012.0021, PMID: 22506599 PMC3342519

[ref10] Barbeyron T. Brillet-Gueguen L. Carre W. Carriere C. Caron C. Czjzek M.. (2016). Matching the diversity of sulfated biomolecules: creation of a classification database for sulfatases reflecting their substrate specificity. PLoS One 11:e0164846. doi: 10.1371/journal.pone.0164846, PMID: 27749924 PMC5066984

[ref11] Becker S. Tebben J. Coffinet S. Wiltshire K. Iversen M. H. Harder T.. (2020). Laminarin is a major molecule in the marine carbon cycle. Proc. Natl. Acad. Sci. U. S. A. 117, 6599–6607. doi: 10.1073/pnas.1917001117, PMID: 32170018 PMC7104365

[ref12] Benner R. (2002). “Chemical composition and reactivity” in Biogeochemistry of marine dissolved organic matter. ed. Hansell, C D. A. A. C. (San Diego, CA, USA: Academic Press, Elsevier Science)

[ref13] Benner R. (2004). What happens to terrestrial organic matter in the ocean? Mar. Chem. 92, 307–310. doi: 10.1016/j.marchem.2004.06.033

[ref14] Benner R. Kaiser K. (2011). Biological and photochemical transformations of amino acids and lignin phenols in riverine dissolved organic matter. Biogeochemistry 102, 209–222. doi: 10.1007/s10533-010-9435-4

[ref15] Bolger A. M. Lohse M. Usadel B. (2014). Trimmomatic: a flexible trimmer for Illumina sequence data. Bioinformatics 30, 2114–2120. doi: 10.1093/bioinformatics/btu170, PMID: 24695404 PMC4103590

[ref16] Boraston A. B. Bolam D. N. Gilbert H. J. Davies G. J. (2004). Carbohydrate-binding modules: fine-tuning polysaccharide recognition. Biochem. J. 382, 769–781. doi: 10.1042/BJ20040892, PMID: 15214846 PMC1133952

[ref17] Bryson S. Li Z. Chavez F. Weber P. K. Pett-Ridge J. Hettich R. L.. (2017). Phylogenetically conserved resource partitioning in the coastal microbial loop. ISME J. 11, 2781–2792. doi: 10.1038/ismej.2017.128, PMID: 28800138 PMC5702734

[ref18] Buchfink B. Xie C. Huson D. H. (2015). Fast and sensitive protein alignment using DIAMOND. Nat. Methods 12, 59–60. doi: 10.1038/nmeth.3176, PMID: 25402007

[ref19] Butler M. K. Den Camp H. J. M. O. Harhangi H. R. Lafi F. F. Strous M. Fuerst J. A. (2007). Close relationship of RNase P RNA in Gemmata and anammox planctomycete bacteria. FEMS Microbiol. Lett. 268, 244–253. doi: 10.1111/j.1574-6968.2006.00597.x, PMID: 17328750

[ref20] Carini P. White A. E. Campbell E. O. Giovannoni S. J. (2014). Methane production by phosphate-starved SAR11 chemoheterotrophic marine bacteria. Nat. Commun. 5:4346. doi: 10.1038/ncomms5346, PMID: 25000228

[ref21] Costa O. Y. A. De Hollander M. Pijl A. Liu B. B. Kuramae E. E. (2020). Cultivation-independent and cultivation-dependent metagenomes reveal genetic and enzymatic potential of microbial community involved in the degradation of a complex microbial polymer. Microbiome 8:76. doi: 10.1186/s40168-020-00836-7, PMID: 32482164 PMC7265232

[ref22] Cottrell M. T. Kirchman D. L. (2000). Natural assemblages of marine proteobacteria and members of the Cytophaga-Flavobacter cluster consuming low- and high-molecular-weight dissolved organic matter. Appl. Environ. Microbiol. 66, 1692–1697. doi: 10.1128/AEM.66.4.1692-1697.2000, PMID: 10742262 PMC92043

[ref23] Cuskin F. Lowe E. C. Temple M. J. Zhu Y. P. Cameron E. A. Pudlo N. A.. (2015). Human gut Bacteroidetes can utilize yeast mannan through a selfish mechanism. Nature 520:388. doi: 10.1038/nature14334, PMID: 25739504

[ref24] Dal Bello M. Lee H. Goyal A. Gore J. (2021). Resource-diversity relationships in bacterial communities reflect the network structure of microbial metabolism. Nat. Ecol. Evolut. 5:1463. doi: 10.1038/s41559-021-01563-4, PMID: 34413507

[ref25] Davis M. P. A. Van Dongen S. Abreu-Goodger C. Bartonicek N. Enright A. J. (2013). Kraken: a set of tools for quality control and analysis of high-throughput sequence data. Methods 63, 41–49. doi: 10.1016/j.ymeth.2013.06.027, PMID: 23816787 PMC3991327

[ref26] Dong H. P. Hong Y. G. Lu S. H. Xie L. Y. (2014). Metaproteomics reveals the major microbial players and their biogeochemical functions in a productive coastal system in the northern South China Sea. Environ. Microbiol. Rep. 6, 683–695. doi: 10.1111/1758-2229.12188, PMID: 25756122

[ref27] Ebrahimi A. Schwartzman J. Cordero O. X. (2019). Cooperation and spatial self-organization determine rate and efficiency of particulate organic matter degradation in marine bacteria. Proc. Natl. Acad. Sci. U. S. A. 116, 23309–23316. doi: 10.1073/pnas.1908512116, PMID: 31666322 PMC6859336

[ref28] Engel A. Thoms S. Riebesell U. Rochelle-Newall E. Zondervan I. (2004). Polysaccharide aggregation as a potential sink of marine dissolved organic carbon. Nature 428, 929–932. doi: 10.1038/nature02453, PMID: 15118723

[ref29] Fahimipour A. K. Gross T. (2020). Mapping the bacterial metabolic niche space. Nature. Communications 11:4887. doi: 10.1038/s41467-020-18695-z, PMID: 32985497 PMC7522980

[ref30] Francis B. Urich T. Mikolasch A. Teeling H. Amann R. (2021). North Sea spring bloom-associated Gammaproteobacteria fill diverse heterotrophic niches. Environ. Microbiome 16:15. doi: 10.1186/s40793-021-00385-y, PMID: 34404489 PMC8371827

[ref31] Garza D. R. Van Verk M. C. Huynen M. A. Dutilh B. E. (2018). Towards predicting the environmental metabolome from metagenomics with a mechanistic model. Nat. Microbiol. 3, 456–460. doi: 10.1038/s41564-018-0124-8, PMID: 29531366

[ref32] Gauthier G. Gauthier M. Christen R. (1995). Phylogenetic analysis of the genera Alteromonas, Shewanella, and Moritella using genes coding for small-subunit rRNA sequences and division of the genus Alteromonas into two genera, Alteromonas (emended) and *Pseudoalteromonas* gen. nov., and proposal of twelve new species combinations. Int. J. Syst. Evol. Microbiol. 45, 755–761. 10.1099/00207713-45-4-755 7547295

[ref33] Gilmore M. C. Cava F. (2022). Peptidoglycan recycling mediated by an ABC transporter in the plant pathogen *Agrobacterium tumefaciens*. Nat. Commun. 13:7927. doi: 10.1038/s41467-022-35607-5, PMID: 36566216 PMC9790009

[ref34] Ginestet C. (2011). ggplot2: elegant graphics for data analysis. J. R. Stat. Soc. A Stat. Soc. 174, 245–246. doi: 10.1111/j.1467-985X.2010.00676_9.x

[ref35] Giovannoni S. J. (2017). SAR11 bacteria: the most abundant plankton in the oceans. Annu. Rev. Mar. Sci. 9, 231–255. doi: 10.1146/annurev-marine-010814-015934 27687974

[ref36] Gowda K. Ping D. Mani M. Kuehn S. (2022). Genomic structure predicts metabolite dynamics in microbial communities. Cells 185:530. doi: 10.1016/j.cell.2021.12.036, PMID: 35085485

[ref37] Gugi B. Le Costaouec T. Burel C. Lerouge P. Helbert W. Bardor M. (2015). Diatom-specific oligosaccharide and polysaccharide structures help to unravel biosynthetic capabilities in diatoms. Mar. Drugs 13, 5993–6018. doi: 10.3390/md13095993, PMID: 26393622 PMC4584364

[ref38] Hallgren J. Tsirigos K. D. Pedersen M. D. Armenteros J. J. A. Marcatili P. Nielsen H.. (2022). DeepTMHMM predicts alpha and beta transmembrane proteins using deep neural networks. bioRxiv. doi: 10.1101/2022.04.08.487609

[ref39] He B. Dai M. Huang W. Liu Q. Chen H. Xu L. (2010a). Sources and accumulation of organic carbon in the Pearl River Estuary surface sediment as indicated by elemental, stable carbon isotopic, and carbohydrate compositions. Biogeosciences 7, 3343–3362. doi: 10.5194/bg-7-3343-2010

[ref40] He B. Dai M. H. Zhai W. D. Wang L. F. Wang K. J. Chen J. H.. (2010b). Distribution, degradation and dynamics of dissolved organic carbon and its major compound classes in the Pearl River Estuary, China. Mar. Chem. 119, 52–64. doi: 10.1016/j.marchem.2009.12.006

[ref41] Hehemann J. H. Truong L. V. Unfried F. Welsch N. Kabisch J. Heiden S. E.. (2017). Aquatic adaptation of a laterally acquired pectin degradation pathway in marine gammaproteobacteria. Environ. Microbiol. 19, 2320–2333. doi: 10.1111/1462-2920.13726, PMID: 28276126

[ref42] Herold M. Arbas S. M. Narayanasamy S. Sheik A. R. Kleine-Borgmann L. A. K. Lebrun L. A.. (2020). Integration of time-series meta-omics data reveals how microbial ecosystems respond to disturbance. Nat. Communicat. 11:5281. doi: 10.1038/s41467-020-19006-2, PMID: 33077707 PMC7572474

[ref43] Hung C. C. Tang D. G. Warnken K. W. Santschi P. H. (2001). Distributions of carbohydrates, including uronic acids, in estuarine waters of Galveston Bay. Mar. Chem. 73, 305–318. doi: 10.1016/S0304-4203(00)00114-6

[ref44] Hyatt D. Chen G. L. Locascio P. F. Land M. L. Larimer F. W. Hauser L. J. (2010). Prodigal: prokaryotic gene recognition and translation initiation site identification. BMC Bioinformat. 11:119. doi: 10.1186/1471-2105-11-119, PMID: 20211023 PMC2848648

[ref45] Jiang W. X. Li P. Y. Chen X. L. Zhang Y. S. Wang J. P. Wang Y. J.. (2022). A pathway for chitin oxidation in marine bacteria. Nat. Commun. 13:5899. doi: 10.1038/s41467-022-33566-5, PMID: 36202810 PMC9537276

[ref46] Jiao N. Z. Herndl G. J. Hansell D. A. Benner R. Kattner G. Wilhelm S. W.. (2011). The microbial carbon pump and the oceanic recalcitrant dissolved organic matter pool. Nat. Rev. Microbiol. 9:555. doi: 10.1038/nrmicro2386-c5 20601964

[ref47] Kieft B. Li Z. Bryson S. Hettich R. L. Pan C. Mayali X.. (2021). Phytoplankton exudates and lysates support distinct microbial consortia with specialized metabolic and ecophysiological traits. Proc. Natl. Acad. Sci. 118:e2101178118. doi: 10.1073/pnas.2101178118, PMID: 34620710 PMC8521717

[ref48] Kits K. D. Sedlacek C. J. Lebedeva E. V. Han P. Bulaev A. Pjevac P.. (2017). Kinetic analysis of a complete nitrifier reveals an oligotrophic lifestyle. Nature 549:269-+. doi: 10.1038/nature23679, PMID: 28847001 PMC5600814

[ref49] Kuang J. L. Huang L. N. Chen L. X. Hua Z. S. Li S. J. Hu M.. (2013). Contemporary environmental variation determines microbial diversity patterns in acid mine drainage. ISME J. 7, 1038–1050. doi: 10.1038/ismej.2012.139, PMID: 23178673 PMC3635239

[ref50] Langmead B. Salzberg S. L. (2012). Fast gapped-read alignment with bowtie 2. Nat. Methods 9, 357–359. doi: 10.1038/nmeth.1923, PMID: 22388286 PMC3322381

[ref51] Lauro F. M. Mcdougald D. Thomas T. Williams T. J. Egan S. Rice S.. (2009). The genomic basis of trophic strategy in marine bacteria. Proc. Natl. Acad. Sci. U. S. A. 106, 15527–15533. doi: 10.1073/pnas.0903507106, PMID: 19805210 PMC2739866

[ref52] Lazar C. S. Baker B. J. Seitz K. Hyde A. S. Dick G. J. Hinrichs K. U.. (2016). Genomic evidence for distinct carbon substrate preferences and ecological niches of Bathyarchaeota in estuarine sediments. Environ. Microbiol. 18, 1200–1211. doi: 10.1111/1462-2920.13142, PMID: 26626228

[ref53] Levy R. Borenstein E. (2013). Metabolic modeling of species interaction in the human microbiome elucidates community-level assembly rules. Proc. Natl. Acad. Sci. U. S. A. 110, 12804–12809. doi: 10.1073/pnas.1300926110, PMID: 23858463 PMC3732988

[ref54] Liu S. T. Baetge N. Comstock J. Opalk K. Parsons R. Halewood E.. (2020). Stable isotope probing identifies bacterioplankton lineages capable of utilizing dissolved organic matter across a range of bioavailability. Front. Microbiol. 11:580397. doi: 10.3389/fmicb.2020.580397, PMID: 33117322 PMC7575717

[ref55] Liu H. Huang L. Tan Y. Zhixin K. E. Liu J. Zhao C.. (2017). Seasonal variations of chlorophyll a and primary production and their influencing factors in the Pearl River Estuary. J. Trop. Oceanogr.

[ref56] Liu Y. Y. Lin Q. Feng J. R. Yang F. M. Du H. Hu Z.. (2020). Differences in metabolic potential between particle -associated and free- living bacteria along Pearl River Estuary. Sci. Total Environ. 728:138856. doi: 10.1016/j.scitotenv.2020.138856, PMID: 32570327

[ref57] Louca S. Parfrey L. W. Doebeli M. (2016). Decoupling function and taxonomy in the global ocean microbiome. Science 353, 1272–1277. doi: 10.1126/science.aaf4507, PMID: 27634532

[ref58] Luis A. S. Basle A. Byrne D. P. Wright G. S. A. London J. A. Jin C. S.. (2022). Sulfated glycan recognition by carbohydrate sulfatases of the human gut microbiota. Nat. Chem. Biol. 18:1032. doi: 10.1038/s41589-022-01132-1, PMID: 35931865

[ref59] Ma X. Johnson K. B. Gu B. Zhang H. Li G. Huang X.. (2022). The in-situ release of algal bloom populations and the role of prokaryotic communities in their establishment and growth. Water Res. 219:118565. doi: 10.1016/j.watres.2022.118565, PMID: 35597219

[ref60] Malmstrom R. R. Cottrell M. T. Elifantz H. Kirchman D. L. (2005). Biomass production and assimilation of dissolved organic matter by SAR11 bacteria in the Northwest Atlantic Ocean. Appl. Environ. Microbiol. 71, 2979–2986. doi: 10.1128/AEM.71.6.2979-2986.2005, PMID: 15932993 PMC1151852

[ref61] Mccarren J. Becker J. W. Repeta D. J. Shi Y. M. Young C. R. Malmstrom R. R.. (2010). Microbial community transcriptomes reveal microbes and metabolic pathways associated with dissolved organic matter turnover in the sea. Proc. Natl. Acad. Sci. U. S. A. 107, 16420–16427. doi: 10.1073/pnas.1010732107, PMID: 20807744 PMC2944720

[ref62] Munoz R. Rosselló-Móra R. Amann R. (2016). Revised phylogeny of Bacteroidetes and proposal of sixteen new taxa and two new combinations including *Rhodothermaeota phyl*. nov. Syst. Appl. Microbiol. 39, 281–296. doi: 10.1016/j.syapm.2016.04.004, PMID: 27287844

[ref63] Myklestad S. M. Børsheim K. Y. (2007). Dynamics of carbohydrates in the Norwegian Sea inferred from monthly profiles collected during 3 years at 66°N, 2°E. Mar. Chem. 107, 475–485. doi: 10.1016/j.marchem.2007.09.002

[ref64] Noell S. E. Barrell G. E. Suffridge C. Morré J. Gable K. P. Graff J. R.. (2021). SAR11 cells rely on enzyme multifunctionality to transport and metabolize a range of polyamine compounds. mBio. 12:e0109121. doi: 10.1128/mBio.01091-21 34425701 PMC8437039

[ref65] Oksanen J. Simpson G. Blanchet F.G. Kindt R. Legendre P. Minchin P.. (2022). vegan community ecology package version 2.6–2 April 2022. Available at: http://CRAN.R-project.org/package=vegan

[ref66] Okuda K. (2002). Structure and phylogeny of cell coverings. J. Plant Res. 115, 283–288. doi: 10.1007/s10265-002-0034-x 12582732

[ref67] Orellana L. H. Francis T. B. Ferraro M. Hehemann J.-H. Fuchs B. M. Amann R. I. (2022). Verrucomicrobiota are specialist consumers of sulfated methyl pentoses during diatom blooms. ISME J. 16, 630–641. doi: 10.1038/s41396-021-01105-7, PMID: 34493810 PMC8857213

[ref68] Orsi W. D. Richards T. A. Francis W. R. (2018). Predicted microbial secretomes and their target substrates in marine sediment. Nat. Microbiol. 3, 32–37. doi: 10.1038/s41564-017-0047-9 29062087

[ref69] Painter T. J. (1983). “4 - algal polysaccharides” in The polysaccharides. ed. Aspinall G. O. (New York: Academic Press), 195–285.

[ref70] Pakulski J. D. Benner R. (1994). Abundance and distribution of carbohydrates in the ocean. Limnol. Oceanogr. 39, 930–940. doi: 10.4319/lo.1994.39.4.0930

[ref71] Parks D. H. Beiko R. G. (2015). “STAMP: statistical analysis of metagenomic profiles” in Encyclopedia of metagenomics: Genes, genomes and metagenomes: Basics, methods, databases and tools. ed. Nelson K. E. (Boston, MA: Springer US), 641–645.

[ref72] Parsons T.R. Takahashi M. Hargrave B. (1984). Biological oceanographic processes. Oxford Oxfordshire; New York: Pergamon Press.

[ref73] Passow U. (2002). Transparent exopolymer particles (TEP) in aquatic environments. Prog. Oceanogr. 55, 287–333. doi: 10.1016/S0079-6611(02)00138-6

[ref74] Pedler B. E. Aluwihare L. I. Azam F. (2014). Single bacterial strain capable of significant contribution to carbon cycling in the surface ocean. Proc. Natl. Acad. Sci. U. S. A. 111, 7202–7207. doi: 10.1073/pnas.1401887111, PMID: 24733921 PMC4034236

[ref75] Peterson A. Soberón J. Pearson R. Anderson R. Martínez-Meyer E. Nakamura M.. (2011). Ecological niches and geographic distributions Princeton, NJ: Princeton University Press.

[ref01] Prahl F. G. Sparrow M. A. Eversmeyer B. Ertel J. R. Goni M. A. (1994). Terrestrial organic carbon contributions to sediments on the Washington margin. Geochim. Cosmochim. Acta. 58, 3035–3048. doi: 10.1016/0016-7037(94)90177-5

[ref76] Reintjes G. Arnosti C. Fuchs B. Amann R. (2019). Selfish, sharing and scavenging bacteria in the Atlantic Ocean: a biogeographical study of bacterial substrate utilisation. ISME J. 13, 1119–1132. doi: 10.1038/s41396-018-0326-3, PMID: 30531893 PMC6474216

[ref77] Reintjes G. Fuchs B. M. Scharfe M. Wiltshire K. H. Amann R. Arnosti C. (2020). Short-term changes in polysaccharide utilization mechanisms of marine bacterioplankton during a spring phytoplankton bloom. Environ. Microbiol. 22, 1884–1900. doi: 10.1111/1462-2920.14971, PMID: 32128969

[ref78] Saier M. H. Reddy V. S. Moreno-Hagelsieb G. Hendargo K. J. Zhang Y. C. Iddamsetty V.. (2021). The transporter classification database (TCDB): 2021 update. Nucleic Acids Res. 49, D461–D467. doi: 10.1093/nar/gkaa1004, PMID: 33170213 PMC7778945

[ref79] Santillan E. Wuertz S. (2022). Microbiome assembly predictably shapes diversity across a range of disturbance frequencies in experimental microcosms. NPJ Biofilms Microbiomes 8:41. doi: 10.1038/s41522-022-00301-3, PMID: 35562363 PMC9106739

[ref80] Sauer D. B. Karpowich N. K. Song J. M. Wang D. N. (2015). Rapid bioinformatic identification of thermostabilizing mutations. Biophys. J. 109, 1420–1428. doi: 10.1016/j.bpj.2015.07.026, PMID: 26445442 PMC4601007

[ref81] Shi Z. Xu J. Li X. F. Li R. H. Li Q. (2019). Links of extracellular enzyme activities, microbial metabolism, and community composition in the river-impacted coastal waters. J. Geophys. Res. Biogeosci. 124, 3507–3520. doi: 10.1029/2019JG005095

[ref82] Sichert A. Corzett C. H. Schechter M. S. Unfried F. Markert S. Becher D.. (2020). Verrucomicrobia use hundreds of enzymes to digest the algal polysaccharide fucoidan. Nat. Microbiol. 5, 1026–1039. doi: 10.1038/s41564-020-0720-2 32451471

[ref83] Smith M. W. Herfort L. Rivers A. R. Simon H. M. (2019). Genomic signatures for sedimentary microbial utilization of phytoplankton detritus in a fast-flowing estuary. Front. Microbiol. 10:2475. doi: 10.3389/fmicb.2019.02475, PMID: 31749780 PMC6848030

[ref84] Smits S. A. Leach J. Sonnenburg E. D. Gonzalez C. G. Lichtman J. S. Reid G.. (2017). Seasonal cycling in the gut microbiome of the Hadza hunter-gatherers of Tanzania. Science 357:802. doi: 10.1126/science.aan4834, PMID: 28839072 PMC5891123

[ref85] Sperling M. Piontek J. Engel A. Wiltshire K. H. Niggemann J. Gerdts G.. (2017). Combined carbohydrates support rich communities of particle-associated marine bacterioplankton. Front. Microbiol. 8:65. doi: 10.3389/fmicb.2017.00065, PMID: 28197132 PMC5281597

[ref86] Sun J. Steindler L. Thrash J. C. Halsey K. H. Smith D. P. Carter A. E.. (2011). One carbon metabolism in SAR11 pelagic marine Bacteria. PLoS One 6:e23973. doi: 10.1371/journal.pone.0023973 21886845 PMC3160333

[ref87] Sun C. C. Wang Y. S. Li Q. P. Yue W. Z. Wang Y. T. Sun F. L.. (2012). Distribution characteristics of transparent exopolymer particles in the Pearl River estuary, China. J. Geophys. Res. Biogeosci. 117:G00N17. doi: 10.1029/2012JG001951

[ref88] Sun C. C. Yue W. Z. Wang Y. S. He W. H. Hong Y. G. Sun F. L.. (2022). Distribution of Coomassie blue stainable particles in the Pearl River Estuary, China, insight into the nitrogen cycling in estuarine system. Front. Mar. Sci. 8:733240. doi: 10.3389/fmars.2021.733240

[ref89] Teeling H. Fuchs B. M. Becher D. Klockow C. Gardebrecht A. Bennke C. M.. (2012). Substrate-controlled succession of marine bacterioplankton populations induced by a phytoplankton bloom. Science 336, 608–611. doi: 10.1126/science.1218344, PMID: 22556258

[ref90] Teeling H. Fuchs B. M. Bennke C. M. Kruger K. Chafee M. Kappelmann L.. (2016). Recurring patterns in bacterioplankton dynamics during coastal spring algae blooms. elife 5:e11888. doi: 10.7554/eLife.11888, PMID: 27054497 PMC4829426

[ref91] Teufel F. Armenteros J. J. A. Johansen A. R. Gislason M. H. Pihl S. I. Tsirigos K. D.. (2022). SignalP 6.0 predicts all five types of signal peptides using protein language models. Nat. Biotechnol. 40:1023. doi: 10.1038/s41587-021-01156-3, PMID: 34980915 PMC9287161

[ref92] Thomas F. Le Duff N. Wu T. D. Cbron A. Uroz S. Riera P.. (2021). Isotopic tracing reveals single-cell assimilation of a macroalgal polysaccharide by a few marine Flavobacteria and Gammaproteobacteria. ISME J. 15, 3062–3075. doi: 10.1038/s41396-021-00987-x, PMID: 33953365 PMC8443679

[ref93] Tremblay L. Benner R. (2006). Microbial contributions to N-immobilization and organic matter preservation in decaying plant detritus. Geochim. Cosmochim. Acta 70, 133–146. doi: 10.1016/j.gca.2005.08.024

[ref94] Tripp H. J. Kitner J. B. Schwalbach M. S. Dacey J. W. H. Wilhelm L. J. Giovannoni S. J. (2008). SAR11 marine bacteria require exogenous reduced Sulphur for growth. Nature 452, 741–744. doi: 10.1038/nature06776, PMID: 18337719

[ref95] Vidal-Melgosa S. Sichert A. Ben Francis T. Bartosik D. Niggemann J. Wichels A.. (2021). Diatom fucan polysaccharide precipitates carbon during algal blooms. Nat. Commun. 12:1150. doi: 10.1038/s41467-021-21009-6, PMID: 33608542 PMC7896085

[ref96] Vollmer W. Joris B. Charlier P. Foster S. (2008). Bacterial peptidoglycan (murein) hydrolases. FEMS Microbiol. Rev. 32, 259–286. doi: 10.1111/j.1574-6976.2007.00099.x 18266855

[ref97] Von Meijenfeldt F. A. B. Hogeweg P. Dutilh B. E. (2023). A social niche breadth score reveals niche range strategies of generalists and specialists. Nat. Ecol. Evolut. 7, 768–781. doi: 10.1038/s41559-023-02027-7 PMC10172124 37012375

[ref98] Wang Y. M. Pan J. Yang J. Zhou Z. C. Pan Y. P. Li M. (2020). Patterns and processes of free-living and particle-associated bacterioplankton and archaeaplankton communities in a subtropical river-bay system in South China. Limnol. Oceanogr. 65, S161–S179. doi: 10.1002/lno.11314

[ref99] Wegner C. E. Richter-Heitmann T. Klindworth A. Klockow C. Richter M. Achstetter T.. (2013). Expression of sulfatases in *Rhodopirellula baltica* and the diversity of sulfatases in the genus *Rhodopirellula*. Mar. Genomics 9, 51–61. doi: 10.1016/j.margen.2012.12.001, PMID: 23273849

[ref100] Wolter L. A. Mitulla M. Kalem J. Daniel R. Simon M. Wietz M. (2021). CAZymes in *Maribacter dokdonensis* 62-1 from the patagonian shelf: genomics and physiology compared to related flavobacteria and a co-occurring alteromonas strain. Front. Microbiol. 12:628055. doi: 10.3389/fmicb.2021.628055, PMID: 33912144 PMC8072126

[ref101] Xing P. Hahnke R. L. Unfried F. Markert S. Huang S. X. Barbeyron T.. (2015). Niches of two polysaccharide-degrading Polaribacter isolates from the North Sea during a spring diatom bloom. ISME J. 9, 1410–1422. doi: 10.1038/ismej.2014.225, PMID: 25478683 PMC4438327

[ref102] Zhang J. Ma K. (2013). Spaa: an R package for computing species association and niche overlap.

[ref103] Zhang Y.-L. Ran Y. (2014). Stable carbon and nitrogen isotopic, amino acids and lignin compositions and geochemical significance of particulate organic matter from the middle and lower reaches of the Pearl River. Geochimica 43, 114–121. doi: 10.19700/j.0379-1726.2014.02.002

[ref104] Zhang Y. Xiao W. Jiao N. Z. (2016). Linking biochemical properties of particles to particle-attached and free-living bacterial community structure along the particle density gradient from freshwater to open ocean. J. Geophys. Res. Biogeosci. 121, 2261–2274. doi: 10.1002/2016JG003390

[ref105] Zhang H. Yohe T. Huang L. Entwistle S. Wu P. Z. Yang Z. L.. (2018). dbCAN2: a meta server for automated carbohydrate-active enzyme annotation. Nucleic Acids Res. 46, W95–W101. doi: 10.1093/nar/gky418, PMID: 29771380 PMC6031026

[ref106] Zhao Z. H. Baltar F. Herndl G. J. (2020). Linking extracellular enzymes to phylogeny indicates a predominantly particle-associated lifestyle of deep-sea prokaryotes. Sci. Adv. 6:eaaz4354. doi: 10.1126/sciadv.aaz4354, PMID: 32494615 PMC7159927

