# Supplementary material for: Polymeric carbohydrates utilization separates microbiomes into niches: insights into the diversity of microbial carbohydrate-active enzymes in the inner shelf of the Pearl River Estuary, China
Source: Front Microbiol. 2023 Jun 21;14:1180321. doi: 10.3389/fmicb.2023.1180321 (PMC10322874; doi:10.3389/fmicb.2023.1180321)
Supplement: Supplementary file 2 [file Presentation_1.pdf]

Supplemental figures

Supplementary figure captions

Figure S1 Compositions of CAZymes at class level (y-axis: red, the surface sediment samples; blue, the bottom water samples; green, the surface water samples； six classes of CAZymes, glycosyl transferases(GTs), glycoside hydrolases (GHs), polysaccharide lyases (PLs), carbohydrate esterases (CEs), carbohydrate-binding modules (CBMs), auxiliary activities (AAs))

Figure S2 Abundance (unit, RPKM) of the dominant GHs in water and sediment, respectively (SF, surface free-living fraction; SP, surface particle-associated fraction; BF, bottom free-living fraction; BP, bottom particle-associated fraction; S, sediment)

Figure S3 Abundance (unit, RPKM) of the dominant AAs, CBMs, and CEs in water and sediment, respectively (SF, surface free-living fraction; SP, surface particle-associated fraction; BF, bottom free-living fraction; BP, bottom particle-associated fraction; S, sediment)

Figure S4 Abundance (unit, RPKM) of the dominant PLs in water and sediment, respectively (SF, surface free-living fraction; SP, surface particle-associated fraction; BF, bottom free-living fraction; BP, bottom particle-associated fraction; S, sediment)

Figure S5 CAZymes diversity (Shannon- index) and glycan niche width (Levins index) at the genus level (FL, free-living fraction; PA, particle-associated fraction; S, sediment)

Figure S6 Different polysaccharides-specific bacterial genera (SF, surface free-living fraction; SP, surface particle-associated fraction; BF, bottom free-living fraction; BP,

bottom particle-associated fraction; S, sediment) (A, Nitrogen-containing polysaccharide (N-glycan)-specific bacterial communities; B, Storage polysaccharide(α-, β-linked glucan and Laminarin)-specific bacterial communities; C, Hemicelluloses, celluloses, and lignin (Hem, cel, and lig)-specific bacterial communities; D, Pectin (Pec) and Alginate(Alg) -specific bacterial communities; E, FRSP-specific bacterial communities)

Figure S7 Competition niches of CAZymes (GH23, 74, and 103) between *Alteromonas* and *CandidatusPelagibacter* on the nitrogen-containing polysaccharides; Green rectangles, bottom samples; Black rectangles, surface samples.

Figure S8 Proportions of phylum to the GH109 and GH33 (A, GH109; B, GH33; FL, free-living fraction, PA, particle-associated fraction)

Figure S9 Contributions to the total Sulfatase abundance at order level (SF, surface free-living fraction; SP, surface particle-associated fraction; BF, bottom free-living fraction; BP, bottom particle-associated fraction; S, sediment)

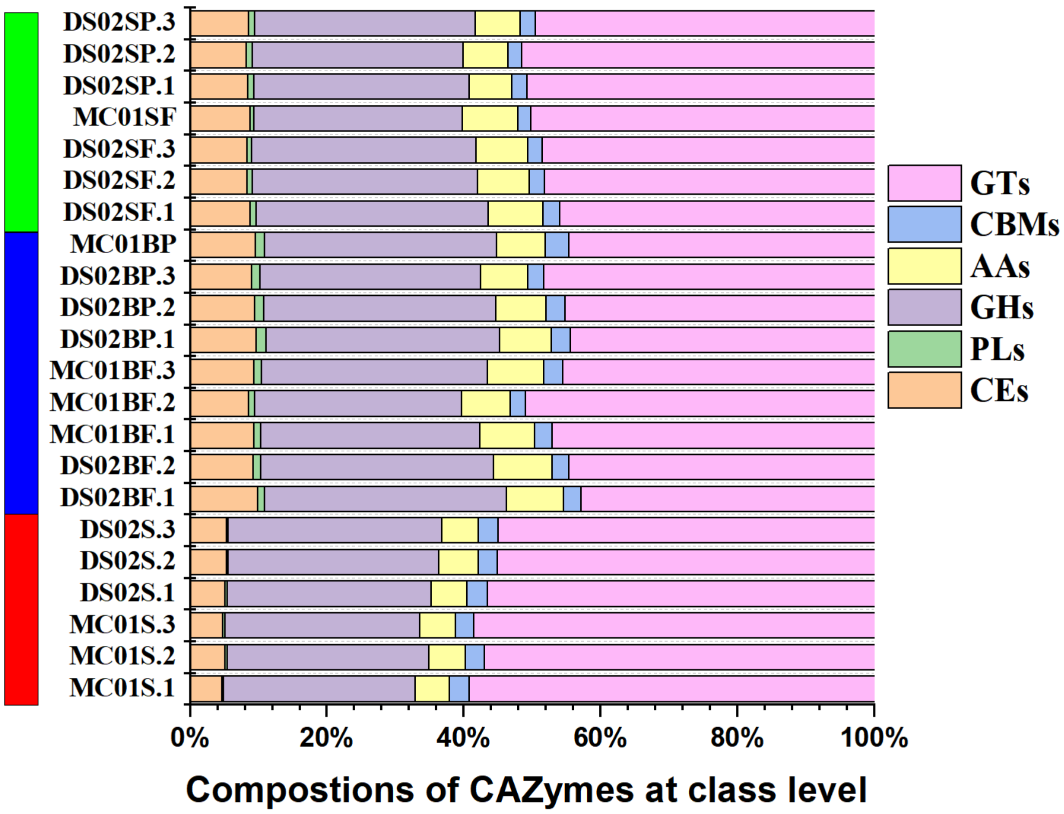
DS02SP.3
DS02SP.2
DS02SP.1
MC01SF
DS02SF.3
DS02SF.2
DS02SF.1
MC01BP
DS02BP.3
DS02BP.2
DS02BP.1
MC01BF.3
MC01BF.2
MC01BF.1
DS02BF.2
DS02BF.1
DS02S.3
DS02S.2
DS02S.1
MC01S.3
MC01S.2
MC01S.1
0%
20%
40%
60%
80%
100%
Compostions of CAZymes at class level
GTs
CBMs
AAs
GHs
PLs
CEs

Figure S1

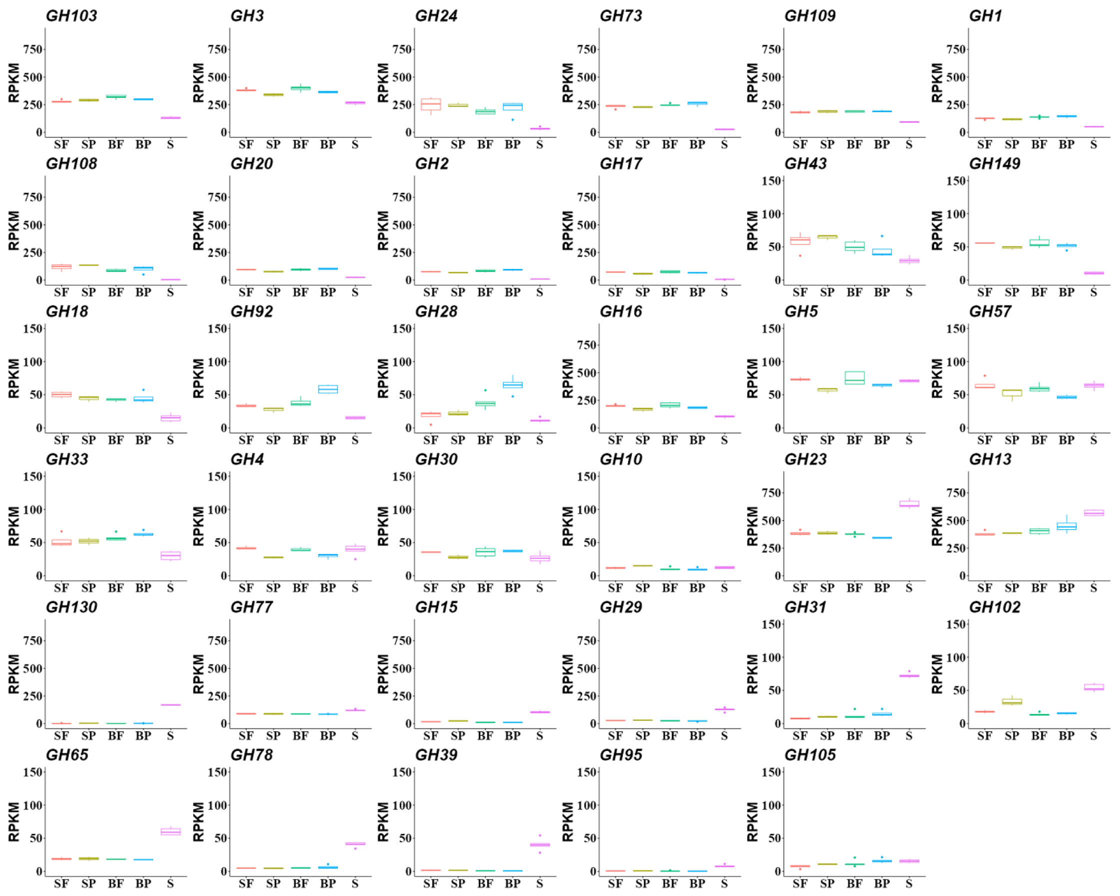
GH103
GH3
GH24
GH73
GH109
GH1
GH108
GH20
GH2
GH17
GH43
GH149
GH18
GH92
GH28
GH16
GH5
GH57
GH33
GH4
GH30
GH10
GH23
GH13
GH130
GH77
GH15
GH29
GH31
GH102
GH65
GH78
GH39
GH95
GH105
RPKM
SF SP BF BP S

Figure S2

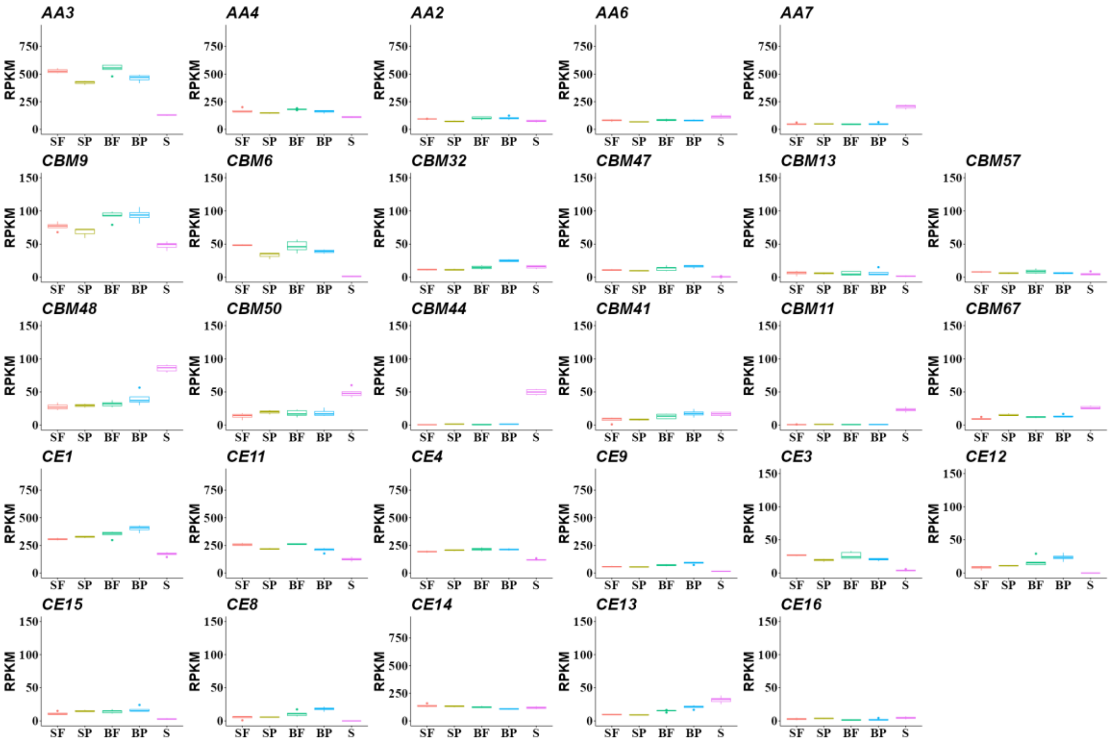
AA3
AA4
AA2
AA6
AA7
CBM9
CBM6
CBM32
CBM47
CBM13
CBM57
CBM48
CBM50
CBM44
CBM41
CBM11
CBM67
CE1
CE11
CE4
CE9
CE3
CE12
CE15
CE8
CE14
CE13
CE16
RPKM
SF SP BF BP S

Figure S3

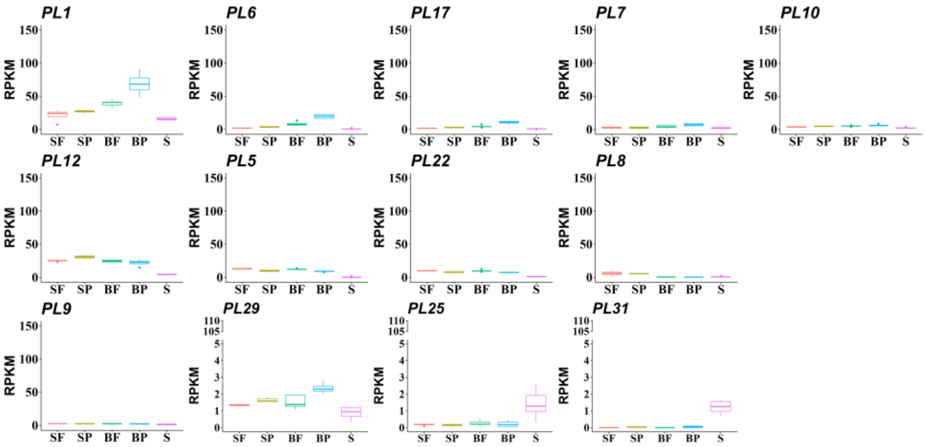
PL1
PL6
PL17
PL7
PL10
PL12
PL5
PL22
PL8
PL9
PL29
PL25
PL31
RPKM
SF
SP
BF
BP
S

Figure S4

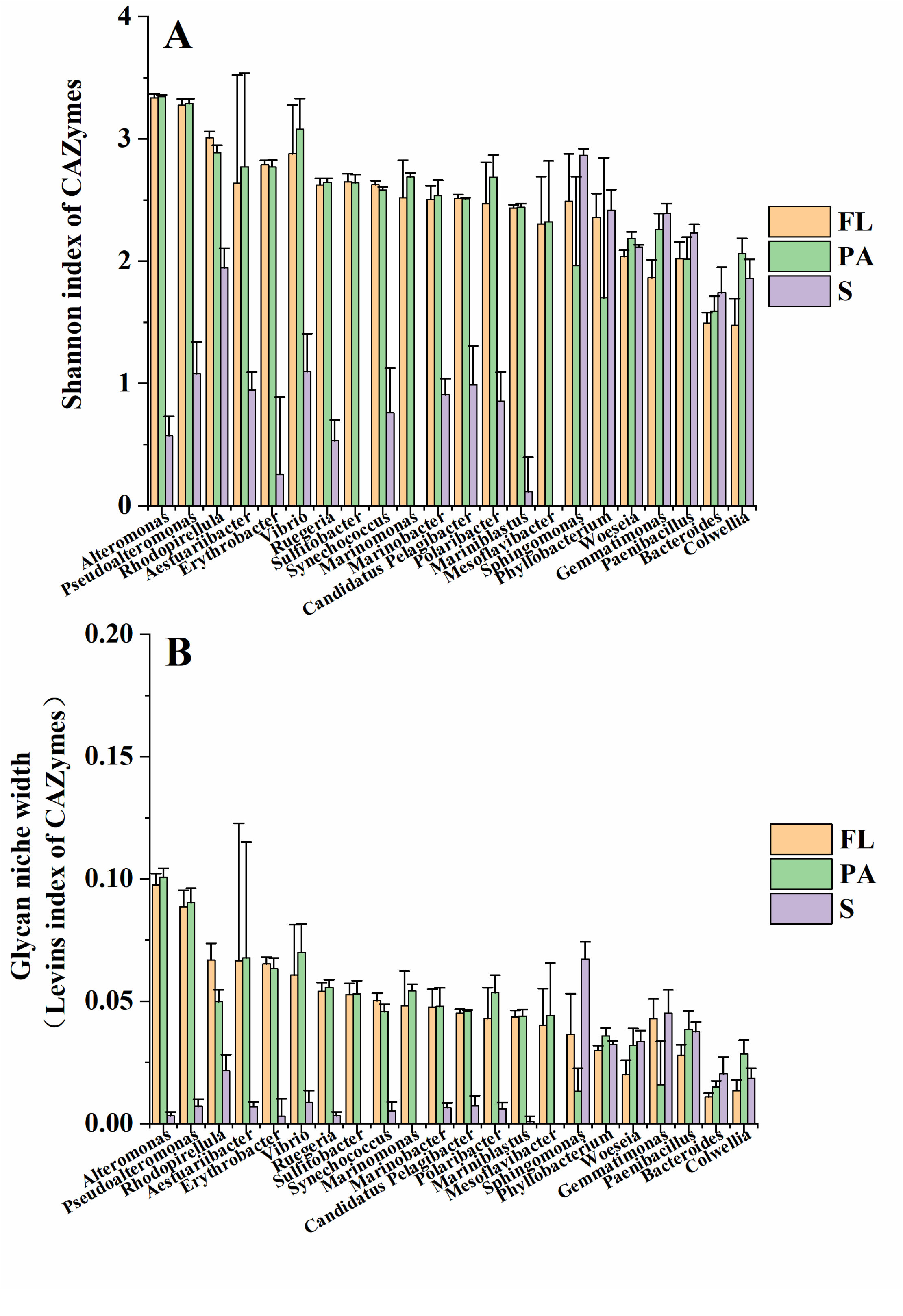
A
Shannon index of CAZymes
4
3
2
1
0
FL
PA
S
Alteromonas
Pseudoalteromonas
Rhodopirellula
Aestuariibacter
Erythrobacter
Vibrio
Ruegeria
Sulfitobacter
Synechococcus
Marinomonas
Candidatus Pelagibacter
Marinobacter
Polaribacter
Mariniblastus
Mesoflavibacter
Sphingomonas
Phyllobacterium
Woeseia
Gemmatimonas
Paenibacillus
Bacteroides
Colwellia
B
Glycan niche width
（Levins index of CAZymes）
0.20
0.15
0.10
0.05
0.00
FL
PA
S

Figure S5

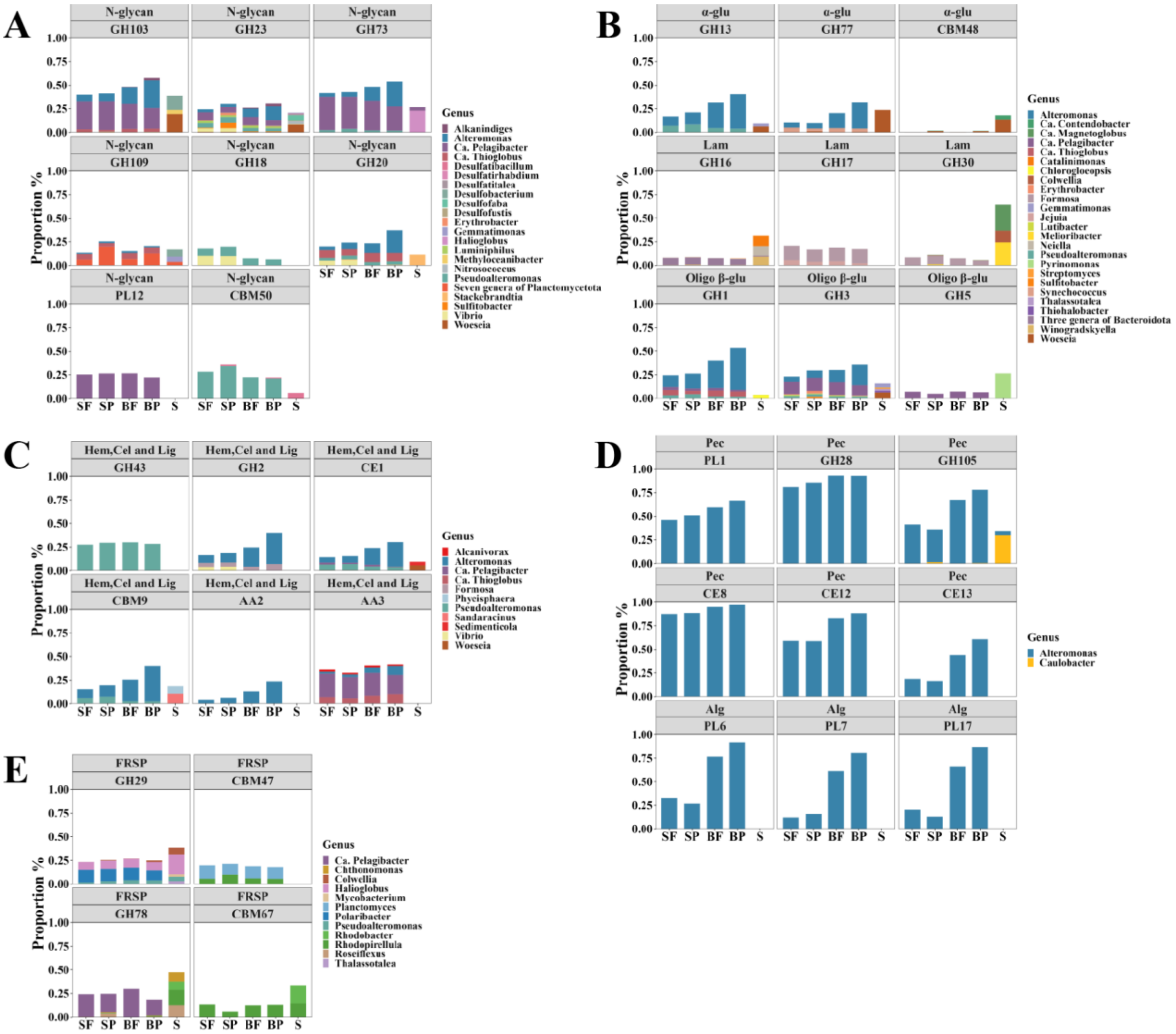
A
B
C
D
E
N-glycan
GH103
GH23
GH73
GH109
GH18
GH20
PL12
CBM50
α-glu
GH13
GH77
CBM48
Lam
GH16
GH17
GH30
Oligo β-glu
GH1
GH3
GH5
Hem,Cel and Lig
GH43
GH2
CE1
CBM9
AA2
AA3
Pec
PL1
GH28
GH105
CE8
CE12
CE13
Alg
PL6
PL7
PL17
FRSP
GH29
CBM47
GH78
CBM67
Proportion %
SF
SP
BF
BP
S
Genus

Figure S6

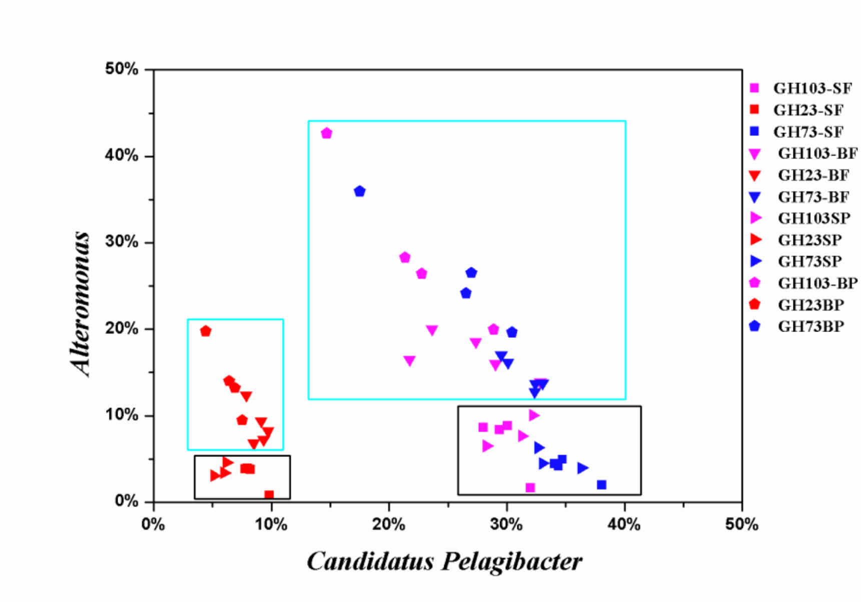

Figure S7

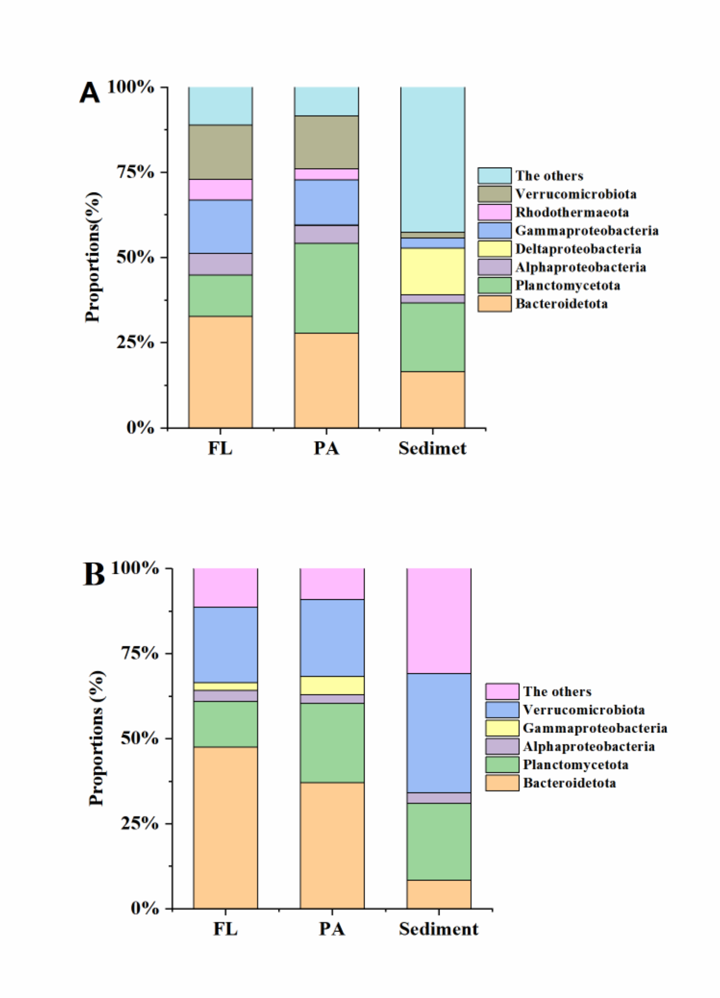

Figure S8

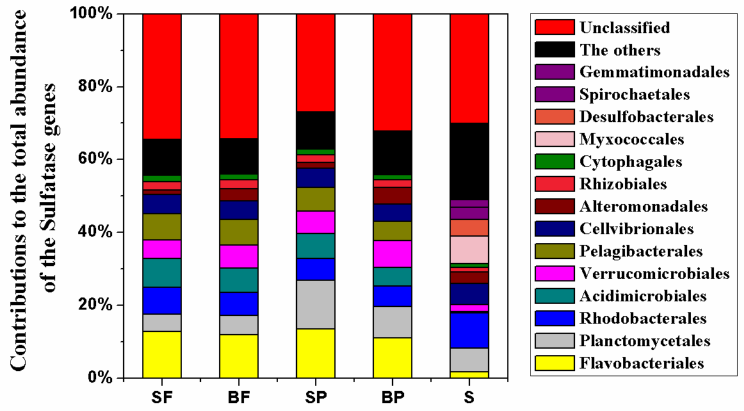

Contributions to the total abundance of the Sulfatase genes
100%
80%
60%
40%
20%
0%
SF
BF
SP
BP
S
Unclassified
The others
Gemmatimonadales
Spirochaetales
Desulfobacterales
Myxococcales
Cytophagales
Rhizobiales
Alteromonadales
Cellvibrionales
Pelagibacterales
Verrucomicrobiales
Acidimicrobiales
Rhodobacterales
Planctomycetales
Flavobacteriales

Figure S9
